# Eye Tracking in Optometry: A Systematic Review

**DOI:** 10.16910/jemr.16.3.3

**Published:** 2023-08-16

**Authors:** Leonela González-Vides, José Luis Hernández-Verdejo, Pilar Cañadas-Suárez

**Affiliations:** Complutense University of Madrid, Madrid, Spain

**Keywords:** Eye-tracking, vision sciences, eye movements, visual technology, optometry, opthalmology

## Abstract

This systematic review examines the use of eye-tracking devices in optometry, describing their main characteristics, areas of application and metrics used. Using the PRISMA method, a systematic search was performed of three databases. The search strategy identified 141 reports relevant to this topic, indicating the exponential growth over the past ten years of the use of eye trackers in optometry. Eye-tracking technology was applied in at least 12 areas of the field of optometry and rehabilitation, the main ones being optometric device technology, and the assessment, treatment, and analysis of ocular disorders. The main devices reported on were infrared light-based and had an image capture frequency of 60 Hz to 2000 Hz. The main metrics mentioned were fixations, saccadic movements, smooth pursuit, microsaccades, and pupil variables. Study quality was sometimes limited in that incomplete information was provided regarding the devices used, the study design, the methods used, participants' visual function and statistical treatment of data. While there is still a need for more research in this area, eye-tracking devices should be more actively incorporated as a useful tool with both clinical and research applications. This review highlights the robustness this technology offers to obtain objective information about a person's vision in terms of optometry and visual function, with implications for improving visual health services and our understanding of the vision process.

## Introduction

Eye-tracking technology was first introduced in the 19th century and was mainly used to analyze reading processes in people. Other applications described have been its use in clinical neuropsychology and in controlling computers through gaze (51; 92).

Recent advances in eye-tracking technology have had an impact in the field of health, including optometry. Today's commercial devices have little to do with their predecessors, and the present is known as the third era of eye trackers with a greater capacity to record and process data (51).

Eye trackers consist of a system with a sensor to detect, measure, and capture eye movements and eye positions. Individual eye movements and what the individual is looking at are tracked through different mechanisms such as using an artificial infrared light source that generates a reflection on the cornea, tracking pupil position, and appearance-based eye tracking. Other hardware systems, such as webcams and smartphones, have also been used (95; TobiiPro, 2015; Valliappan et al., 2020). These devices also work with special software to process the data and interpret the information obtained.

A multitude of techniques exists to record eye movements, including mirror reflection systems, electrooculogram systems, photoelectric and video-based limbus tracking, sclera coils, canthus and corneal bulge tracking, tracking retinal features, dual Purkinje imaging, dark and bright pupil tracking, pupil and corneal reflection, laser-based pupil and iris tracking, video-based tracking of artificial markers, and the most common method, pupil center corneal reflection (51). These techniques are employed by devices such as screen-based eye trackers or wearable eye trackers to collect information.

Eye tracking devices enable the monitoring and recording of eye movements, providing valuable information that can be extracted as raw data samples, including pupil size, pupil position, corneal reflections, fixation and saccade velocities and accelerations, gaze vectors for each eye, and gaze points. Other metrics, such as fixations, saccades, post-saccadic oscillation, smooth pursuit, microsaccades, tremor, and drifts, can be derived from these gaze-related event metrics (32; 34; 51; 64; 95; 107; 110; 141).

Eye-tracking metrics can facilitate the acquisition of relevant information regarding various aspects of human behavior. Accordingly, eye trackers are used in cognitive psychology, to analyze human-computer interactions, and in marketing, psycholinguistics, neurolinguistics, and sports science, among others (51; 103). The basis for these applications is that eye movements provide different levels of information, including gaze properties, eye properties, perception properties, characteristics of cognitive processes, and even opinions and ideas about people’s reasoning and clinical aspects of different pathologies (3; 7; 33).

The data obtained from these systems can be used for vision assessment, as they offer precise information and metrics on ocular behavior. Eye-tracking technology is also employed in vision analysis instruments that have frequent applications in ophthalmology. Examples are eye trackers associated with optical coherence tomography (OCT), scanning laser ophthalmoscopy and microperimetry.

While eye trackers provide excellent data on certain eye movements, they are highly sophisticated and can be cumbersome to transport (96) limiting the possibilities of modifying the stimuli received or analysis conducted in real-world contexts. In addition, as occurs with the microperimeter, they only allow for monocular tests. As in most individuals the eyes move congruently, a monocular analysis may not offer a comprehensive understanding of human behavior in real-life situations, such as under conditions of low vision.

To circumvent this limitation, new eye tracker devices have been developed for clinical optometry applications. Among these instruments, we should mention screen-based devices and special glasses designed to facilitate manipulation of the stimulus received, the adaptation of tasks, and the selection of eye movements to analyze. These characteristics of eye trackers expand their possibilities to include adjusting instrument calibration or the presentation of stimuli to analyze visual function in specific populations such as children or persons with low vision.

The use of this technology also requires being aware of factors enabling the collection of high-quality data. These include the device's characteristics, sampling rate, accuracy average, the eye-tracking mechanism, and eye-tracking setups or methods of testing. In addition, the importance of calibration, control of head movements, and the characteristics of the environment, such as lighting, temperature and noise, must be considered.

This is why it is important to examine how, up until now, this technology has been used in optometry. Moreover, given that eye trackers have reached a new level of technological readiness, it needs to be established whether these devices and their optometric applications are ready for use in clinical practice.

So far, several review studies have examined the evolution of eye-tracking technology. In 2017, one such review examined the use of microperimetry to assess visual function in age-related macular degeneration. The authors of this study highlighted the benefits of incorporating an eye-tracker in a microperimetry system to correct the position of the stimulus for changes in fixation (23).

In 2020, another study addressed the use of eye tracking in ophthalmology, indicating that this technology is used in modern imaging instruments for patient assessment and imaging diagnostics. This technology also seems to have applications in ophthalmic and refractive surgery (96). However, literature reports of optometry research in general have not discussed the use of this technology in depth, although it has been much used in clinical practice.

## Methods

### Design

This systematic review was designed to assess the benefits of eye tracking in the field of optometry. We used the Preferred Reporting Items for Systematic Reviews and Meta-Analyses (PRISMA) to describe the information collected and registered our study protocol with PROSPERO (registration number 364762). The flow of the PRISMA search is detailed in Fig. 1. RevMan software (version 5.4, Cochrane, UK) was used to record information.

### Search Strategy

The search was conducted in January 2022 and rechecked in July 2022. Titles, abstracts, and bodies of texts were searched in the databases Scopus, Web of Science, and PubMed using the descriptors: ((eye track**) AND (visual acuity)) AND (eye movements) AND (assessment) NOT ((drugs) NOT (psychology). Visual acuity was included as visual function is usually related to visual acuity. This descriptor also helped restrict our search to studies relevant to optometry.

### Study Selection

The following inclusion and exclusion criteria were consistently applied throughout the process: 1) papers published between 2017 and 2022 were included to obtain the most recent information; 2) titles and abstracts related to the field of vision; 3) studies conducted in humans; 4) studies using eye-tracking devices as part of their methodology; 5) papers published in English; and 5) conference papers and journal articles. The review excluded editorials, reviews, studies on animals and reports on cognitive function or psychology-related topics.

Inclusion criteria were applied and each title and abstract was screened by two independent reviewers. When a title or abstract provided insufficient information, the reviewers discussed and made decisions to resolve any disagreement. This served to avoid the risk of missing important information and potentially eligible articles.

### Data Extraction and Quality Assessment

The reviewers independently reviewed full texts and extracted important data in a collection form. The data extracted for each study were year of publication, main objective, subjects, device characteristics, and metrics/paradigms used. As the review sought to identify the areas of optometry in which eye-tracking systems are used, the parameters extracted could vary from one study to another.

Study quality was assessed based on the following criteria: number of participants, method implemented, reporting of the characteristics of the eye tracker used, and the way in which it was employed.

### Statistical Analysis

Given the nature of this systematic review and the lack of quantitative data provided by many of the studies included, the results of our review are presented in narrative form.

## Results

In the search and selection process, 1340 reports were identified, of which 714 remained after removing duplicates. Next, the selected articles were screened according to their publication date, language, title, and abstract, leaving 181 candidate reports. Six papers were excluded because they used animals in the studies. The remaining 175 full-text articles were assessed for eligibility, leaving only 141 reports after discarding 34 as they did not exactly relate to the topic.

The initial database search yielded 1340 reports on the use of eye trackers in the field of vision since 1968 (Fig. 2). In the past ten years, studies related to the use of eye tracking devices in optometry have undergone an exponential increase, averaging at around 17 articles per year. The year 2018 stands out from the rest as there were 24 scientific publications on the topic.

**Figure 01.**
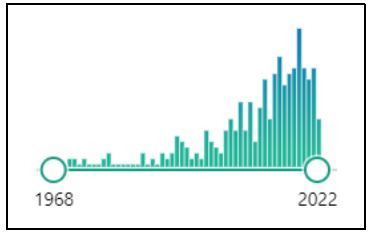
PubMed diagram of publications about eye trackers in vision science by year.

### Eye Trackers in the Field of Optometry

After an exhaustive review of all 141 selected reports, these were grouped by area of application in the field of optometry as described below. In Table 2, the information is organized by area of application, main metrics, devices used, benefits of eye tracking, and methodological aspects.

#### Nystagmus

Nystagmus is an involuntary oscillatory movement of the eyes that can affect a person's visual acuity (104). This disorder was analyzed by eye tracking in four of the articles reviewed here. Different types of nystagmus were examined such as voluntary flutter during ophthalmoscopy (Norouzifard et al., 2020; 123) or optokinetic nystagmus (Norouzifard et al., 2020). Some authors highlighted the capacity of eye-tracking devices to adequately assess individuals with this condition (104).

#### Visual Acuity

Six of the articles reviewed described studies in which visual acuity was examined as the ability to see details using eye-tracking devices. Four of the studies conducted an analysis of dynamic visual acuity (Ağaoğlu et al., 2018; Chen & Yeh, 2019; Domdei et al., 2021; Palidis et al., 2017) while two investigations focused on behavior, visual control, and detection of targets in situations of reduced visual acuity (Freedman et al., 2018, 39).

#### Visual Field

Eye-tracking devices are also used in visual field exams. Nine publications were found on this topic describing studies in which eye movements were used to grade visual health (80) and classify visual field loss (48; 83). The authors of these studies also explored how vision and eye movements function in a monocular or binocular way when there is central visual field involvement. In individuals with hemianopia, the use of these devices to determine the preferential retinal locus was described in subjects with a macular disorder (15; 16; 83; 113; 137).

#### Amblyopia/Strabismus/Vergences

Seven of the articles reviewed addressed amblyopia, strabismus and vergences. In one study, anomalies in monocular and binocular motility were analyzed in children with amblyopia (85). Eye-tracker devices were also used as a resource to detect strabismus, mainly characterizing skills for fixation stability (7; 61; 142) and visual searching (4; 108; 127).

Three of the studies reviewed focused on the topic of exotropia as one of the most common types of strabismus. In these studies, the factors considered were exophoria, intermittent exotropia (3; 33), and longitudinal changes in slow visual searches in subjects with this type of exotropia undergoing surgery (79). Ocular incompatibility and dominance were also addressed by some authors (3).

Nine of the studies identified the use of eye-tracking devices to analyze visual functions. These included characteristics such as convergence, divergence, accommodation, and stereopsis. Eye movement data were collected when taking measurements at the near-point of convergence or vergence (38; 63; 86; 97). Some studies even used technology to perform automated measurements of phorias and heterophorias and determine the influence of age on adaptation to disparity (8; 41; 78, 77, 76).

#### Surgery

Three articles examined ocular motility in relation to surgery: one study after strabismus surgery (91), another assessed the use of eye trackers in small-incision lenticule extraction (SMILE) procedures (101), and finally, Coletta and colleagues examined the impact on eye motility of refractive surgery (29).

**Figure 02.**
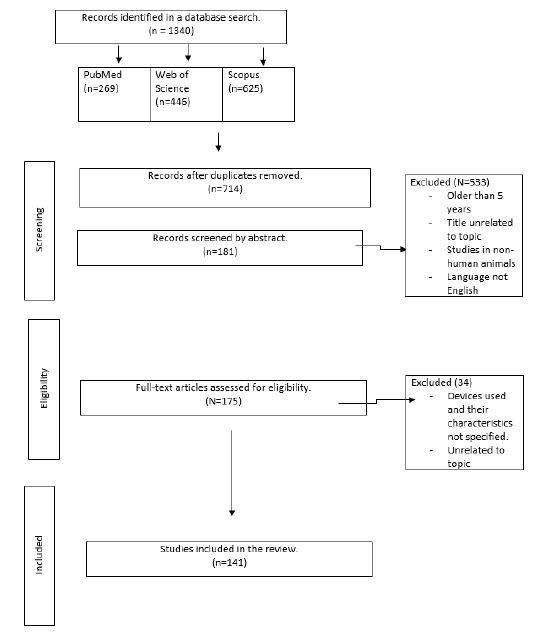
Preferred Reporting Items for Systematic Reviews and Meta-Analyses (PRISMA) flow chart.

#### Technology/Visual Equipment/Virtual and Augmented Reality

Some technological devices, such as video games and virtual and augmented reality systems, incorporate gaze-tracking systems that enable the capture of gaze data. Our review found 18 articles addressing topics related to hardware or software used to improve techniques such as calibration methods, foveated rendering, or the use of portable devices (5; 26; Esfahlani et al., 2019; 40; 59; Kim et al., 2019; Love et al., 2021; Pundlik et al., 2019; 104). These studies highlight a need for more robust eye trackers for use in different instruments including OCT and microperimetry devices (27; 35; 49; 50; 68; 75; 84).

#### Ocular Pathology/Low Vision

Nineteen publications documenting the study of ocular disorders were found. The main diseases examined with eye-tracking technology were glaucoma, Stardgart's disease, age-related macular degeneration, diabetic macular edema, and sequellae of concussion. These investigations characterized and examined in detail oculomotor aspects, fixation stability and binocular fixation (2; 10; 14; 17; 42; 43; 44; 57; 65; 67; 69; 112; 116; 120; 125). Aspects related to visuospatial orientation and visual comfort were also analyzed (14; 43; 65; 71). One study was designed to explore how binocular eye movements behave in the presence of scotoma in terms of binocular vision and contrast sensitivity in peripheral vision (6). Other authors used an eye tracker to analyze the eye movements of drivers diagnosed with glaucoma (70).

#### Assessment/Diagnosis/Rehabilitation/Training

Most eye-tracking research effort was found centering on the assessment, diagnosis, and treatment of various ocular conditions. We therefore selected 24 articles describing the use of ocular motility parameters for such purposes. In these studies, eye-tracking devices were employed to assess color vision (119), contrast sensitivity (122), and visual function (136). Ocular motility was used as a biomarker of visual function beyond visual acuity (Brodsky & Good, 2021; Liston et al., 2017; 72), to develop simple tests to assess slow-to-see behavior in children (132), to evaluate attention in children with high-visual acuity (98), and to analyze visual comfort and visual acuity changes in terms of microsaccades (114). Eye tracking has also been used to assess non-pathological aspects of eye disorders such as visual fatigue (81; 106; 109; 122; 131; 133; 138), and to identify the preferred retinal locus when there is scotoma in persons with macular degeneration (140, 139). These publications also report on the use of eye trackers to restore visual capacity, and to train visual fields and visual searching(22; 12; 24; 53; 80; 90; 122; 130).

#### Refractive Error

Two of the studies reviewed examined ocular motility according to refractive error (31; 88).

#### Reading in Optometry Assessment

Nine of the reports reviewed analyzed patterns of ocular motility during reading in both healthy subjects and in those with conditions such as nystagmus (36) or delayed reading (129). These studies were conducted mainly in children or individuals with visual field loss (102). Stimuli used were short, long, and dynamic texts, and blue light filters (105). Some authors also compared rapid serial visual presentation and horizontal scrolling text (21; 54; 82; 91). In other studies, only the benefits of the use of eye trackers during reading were explored (134).

#### Sports Vision/Locomotion

Another vision field that has been growing in recent years is sports vision, but only one article dealing with this topic was identified. The authors of this report analyzed changes produced in visuomotor behavior in children during training in combat sports (60).

#### Oculomotor Deficits/Oculomotor Responses

Two of the articles reviewed focused on oculomotor responses. One study addressed oculomotor behavior in response to changes at the vestibular level, and the other study was designed to compare oculomotor deficits in adopted and non-adopted children from an unspecified region of Europe (94; 135).

#### General Eye Movements

Finally, twenty-one of the articles included in our review examined ocular motility in general without focusing on any given population. While these reports cannot be assigned to any of the previous sections, their findings have contributed to the field of optometry. Some of the studies described were designed to determine how much time one needs to fixate during different tasks (18; 20; 25; 56; 62; 99; 111), whether fixation stability leads to reduced head movements in people with Argus II (22), or whether this stability differs between central and peripheral vision (100).

These studies also analyzed metrics related to different aspects of saccades, such as saccadic rhythmicity (9; 93; 115), presaccadic motion ( 66), patterns of saccades (13), characteristics of small saccades and microsaccades (37; 87), dynamic perisaccades (55), smooth pursuit (45; 46), and visual perception (89; 128). Some articles highlighted data on disparity and deviations between the eyes and different positions of gaze (16; Barraza-Bernal, Rifai et al., 2017b; 73; 86; 97; 135).

### Methods Used

#### Main Devices and Their Characteristics.

Eye tracking devices use different methods of collecting information on eye positions. In most of the studies reviewed, this was pupillary corneal reflection. In Appendix 1, the devices used by area of application are listed. Four eye tracker brands emerged as most used in the field of optometry: Eyelink 1000 (SR Research, Ontario, Canada) (n=37), those of the company Tobii (TobiiTechnology, Stockholm, Sweden) (n=36), Dual Purkinje eye tracker (Fourward Technologies) (n=5), and the SMI eye tracker (SensoMotoric Instruments GmbH, Teltow, 95 Germany) (n=7).

Some of the eye trackers are incorporated in other technological systems used for vision assessment such as microperimeters (n=2) and tracking scanning laser ophthalmoscopes (n=3). Across all references, these devices have an imaging capture frequency between 60 Hz and 2000 Hz, and are based on the infrared light technique. Both head-mounted and screen-based devices have been employed, depending on whether the exam setting is real-world or controlled environment, respectively.

**Table 2.**
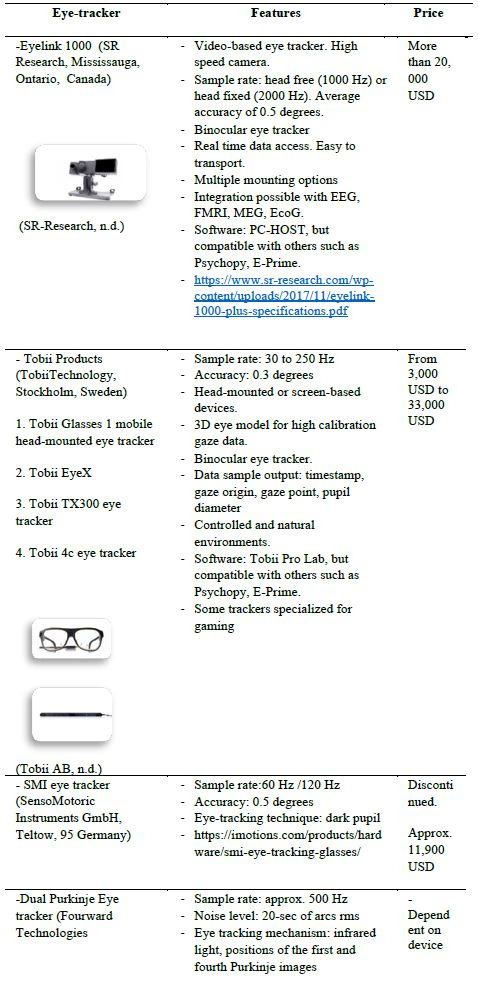
Most commonly used eye-tracker systems.

#### Stimuli Setups and Recordings

Owing to the diversity of study populations and objectives, stimulus setups varied widely in terms of color, size, and shape. Setups ranged from a simple black fixation point in primary gaze position or in different positions on the screen, to smooth pursuit stimuli and cartoon videos for pediatric populations (26; 94; 129), images of real-life situations (5), or standardized tests assessing reading or ocular motility (102; 119; 137). Some stationary and dynamic stimuli were created by the researchers mainly using programs like Matlab and Python.

Each experiment started with some calibration procedure. These ranged from using two dots in two different positions, to as many as nine dots. Only a few research groups reported on the accuracy considered acceptable for calibration (1º of accuracy) (3; 33).

Additionally, the distance between the individual and stimulus varied from 20 cm to 6 m, the average distance in most experiments being 55 cm. For each experiment or trial, durations varied as they depended on the individual’s reaction.

#### Main Metrics Used and Statistical Analysis

According to the data obtained, the main metrics used in the studies as a source of information were those related to fixations (n=60), saccadic movements (n=41), eye position (n=25), smooth pursuit (n=13), and microsaccades (n=10). For each motility measure, the factors analyzed were number and total average, total time, average time, frequency, amplitude of motion, total time in activity, and reaction time. On occasion, data were analyzed both monocularly and binocularly.

Event-related measures were mainly used to locate any important event (metrics) while recording the timeline. In this process, eye movements were first recorded and then metrics were analyzed. In a few reports, algorithms were implemented to obtain quantitative data. The most used procedure was the bivariate contour ellipse area (BCEA) method, which served to calculate fixation stability.

Only some reports indicated the type of research conducted: prospective experimental cohort study (43), comparative study (121), prospective study (130), longitudinal study (65), cross-sectional studies (49; 57; 67; 82; 85), and case-control study (42). Twenty-seven of the articles reported having established a control group with which to compare data from subjects with eye disease.

In general, the articles mentioned several modes of statistical data treatment. Most reports provided descriptive statistics (mean, median, standard deviation, interquartile range), and described the use of comparison and correlation tests on their datasets, and dependent and non-dependent groups for parametric and nonparametric data (Appendix 1).

## Discussion

According to our database search, eye-tracking devices have been used for scientific research in the field of optometry since 1968. The year 2018 stands out as the year in which there was the largest number of publications related to this topic. Within the field of optometry, the use of these devices has been described in at least 12 different areas.

Our comparison of the different studies identified and reviewed was hindered by the diversity of data handled, mainly because of the different populations, eye-tracking devices, and metrics involved.

### Main Areas of Application

Among the main application areas of eye trackers (optometry device technology, and assessment, treatment, and analysis of eye conditions), our review revealed the diagnosis and treatment of different eye conditions as a constant interest in optometry. Consistently, in recent years, eye-tracking technology has been used to improve vision assessment, expand vision care services, for example, to include applications of eye trackers in telemedicine, and even allow for the early detection or and/or prevention of certain eye conditions (30). For these purposes, eye-tracking programs are useful tools as they can be easily integrated into commonly used technologies such as tablets, cell phones, and computers (58; 59).

### Devices Used and Their Characteristics

The most common characteristic of the eye-tracking devices employed in the studies reviewed were infrared light technology, involving a capture frequency greater than 60 Hz, although most exceeded 125 Hz (51). The characteristic features of the four most commonly used devices are compared in Table 2. Devices marketed by Tobii and Eyelink seem to be the preferred systems yet their availability in some institutions is somewhat limited by their high cost.

The mechanism of action of most devices reported on was the corneal reflex technique. However, some research groups used eye trackers based on pupil-corneal reflection to obtain more static measurements, such as fixations, as this technique is considered ineffective to study the dynamics of eye movements or dynamic saccadic movements (52; 118).

Most eye trackers were screen-based devices. These allow for a more controlled environment, the presentation of specific stimuli on screen, and even the incorporation of a chinrest, which is recommended to control head movements (60; 123).

Some studies reported on the use of head‑mounted trackers. Such devices are useful for taking measurements in different environments and even during persons’ daily activities or real-life situations (1; 25; 28; 39; 40).

It should be mentioned that almost all the reviewed studies used experimental setups such that the devices used were not designed as optometric diagnostic systems. This meant that extensive individual analysis of each person may not have been possible. Currently, only especially manufactured optometric devices have wide applications in clinics.

### Main Metrics Used

Many of the studies reviewed were designed to analyze fixations, saccadic movements, smooth pursuit, microsaccades, and pupil variables, as the typical ocular movements used to generate metrics (51). Most reported data were obtained by segmenting the experiment into distinct tasks or events. This allowed for an improved analysis of ocular behavior at specific moments and made it possible to assess the total number of fixations or saccades produced and their durations and amplitudes, visual search patterns, smooth pursuit, and reaction time.

### Methods and Statistical Aspects

After analyzing certain methodological aspects, we found that only 10 articles provided information on the type of study conducted. Most studies were cross-sectional and designed to examine the prevalence and diagnosis of patient conditions such as nystagmus, low vision, and strabismus, among others. In most reports, the sample size estimation method used was unclear, and an arbitrary size was mentioned (25). Only one group reported on the use of Cochran's formula (36).

Subject population ages varied from children to adults, revealing the wide applicability of eye trackers. As these are automatic capture devices for which participation or active response of the subject is not always required, they can be employed to assess behavior and visual function in newborn children (61; 90) or older adults (26; 27; 46; 68; 69, 70; 72; 113; 115; 139).

In some papers, the specific use of the metrics collected or even the eye-tracking paradigm applied were not specified. It is important to study a specific phenomenon and design the experiment properly (51). Only nine studies indicated in their methodology the eye-tracking paradigm addressed (7; 13; 14; 17; 65; 89; 98; 128; 133), especially those assessing eye movements in general, in which the goal was to analyze ocular behavior.

Unlike other studies where eye trackers serve to learn about emotional or cognitive aspects, optometry studies may mainly involve the use raw eye movement data. Many of the studies reviewed, however, had limitations in reporting quality (quality information, statistical power, calibration accuracy, and data loss, among others). Moreover, some did not clearly specify design characteristics and environmental factors (such as device model, frequency of image capture, position, illumination, and temperature). Only one article mentioned having done a pilot study (89), as is usually recommended for eye-tracking studies.

Most articles centered on ocular pathologies and low vision reported a large volume of data on visual function. In addition, authors mentioned calibration changes made to ensure subjects could see the stimulus presented. This is important as in this specific area of optometry, extensive subject characterization in terms of visual acuity, contrast sensitivity, or visual field is needed because eye movements can be affected by these factors (19). Other factors that may need to be specified depending on the main study objectives are pupil size, the number and duration of fixations, saccadic movements and regressions, and eye video, as well as the type of stimulus used, its size and color, the conditions of the space and screen features (124).

With regard to the statistical treatment of the data obtained, besides providing descriptive statistics such as means, medians, and standard deviations, non-parametric tests were mainly used (Mann-Whitney U, Wilcoxon test, Kruskal Wallis test, Friedman test) as most studies were based on non-representative samples. Three articles patently explained the limitations of eye trackers in their studies, such as calibration problems (31; 80), reflection from the surface of lenses (31), the use of glasses, and sample size (134).

Further, although the quality of the evidence provided varied between articles, fourteen studies directly analyzed the usefulness of eye trackers and offered relevant data regarding the validity of their clinical application. Some authors also emphasized the effectiveness and potential of eye trackers, especially for the identification and rehabilitation of patients with ocular disorders, specifically those with visual field loss, through the use of gaze data (67; 72; 99; 139).

Our review identified a need to continue promoting research in this area and to replicate studies with sufficient methodological weight to extrapolate results to the general population.

### Technological Readiness

Technology Readiness Level (TRL) is a systematic method designed to grade the maturity level of a given technology. It consists of nine levels:

TRL 1: Basic principles observed

TRL 2: Technology concept formulated

TRL 3: Experimental proof of concept

TRL 4: Technology validated in the laboratory

TRL 5: Validated in a relevant environment

TRL 6: Technology demonstrated in a relevant environment

TRL 7: System prototype demonstration in an operational environment

TRL 8: System completed and qualified

TRL 9: System proven in operational environment (74).

Many of the devices employed in the studies reviewed, such as those manufactured by Tobii® and Eyelink®, are already marketed and employed in different contexts, including real-life scenarios. However, according to a TRL analysis of the applications of eye-tracking technology in optometry, most studies fulfill the criteria of the first four levels. These levels are related to proof of concept, whereby research is conducted in a laboratory setting in a controlled environment to emphasize that this new technology is valid. Results are presented as measures of specific parameters or metrics.

While several studies have attempted to utilize eye movement measurements in real-world scenarios, such as face recognition in sports or driving (60; 70; 132), these efforts have only yielded basic metrics, rather than new software or hardware applying eye-tracking technology.

One such study reported on the use of a DIVE device (94), a product based on eye-tracking technology that is currently marketed for clinical optometry practice. This device is specifically designed to assess visual function and ocular motility in children. We suggest that in consequence this technology could be graded as TRL level 8 as it is relatively new to the market and not yet widely used.

Eye-tracking devices are still expensive, and their use also requires precision and basic knowledge to avoid handling difficulties leading to erroneous data. In consequence, their clinical applicability in optometry is relatively incipient, and the studies available can be described as pre-clinical. Notwithstanding, the results of some of the studies reviewed here reveal the potential of eye-tracking technology to develop preliminary eye movement models in patients (40), as well as vision tests to investigate the impacts of visual training (26). This suggests that this technology can be applied to various stages of clinical practice, including assessment, intervention, and treatment monitoring.

According to the results of our review, eye-tracking systems are set to have an impact on optometry practice, but it is crucial that we continue to work on new ideas to improve on the readiness of this technology, including the development of standardized, simplified validated procedures.

As limitations of our review, we should mention the unavailability of data emerging from optometry eye-tracker studies. This is not the case in other fields such as psychology and neuroscience. This review, however, provides a comprehensive overview for optometry professionals wishing to investigate or apply eye-tracking systems in clinical practice. By providing information on the devices and metrics used, we hope to encourage new lines of research with practical implications for patient care.

## Conclusions

The use of eye-tracking devices in optometry has exponentially grown over the past ten years, such that this technology is now used in at least 12 areas of optometry and rehabilitation, but mostly in the areas of technology, and the assessment, treatment, and analysis of ocular disorders.

Eye trackers use data from the visual system and ocular motility information to record the visual behavior of individuals of any age both in natural and controlled environments, thus expanding possible applications from laboratory settings to clinical practice.

We propose that these tools should be incorporated more actively in optometry, both in research and clinical applications, as they offer robust objective information about an individual’s vision in terms of optometry and visual function, with the ultimate goal of improving visual health services and our understanding of the vision process.

For the use of eye trackers in clinical practice, it is important that professionals have precise knowledge of the characteristics of the software and hardware used to ensure the acquisition of valid data while considering their limitations. It is especially important that new procedures become standardized, simplified in their application, and validated. It is clear that eye-tracking systems will gradually gain popularity in the optometry field, not only for assessment but also for treatment and training.

### Ethics and Conflict of Interest

The authors declare that the contents of this article are in agreement with the ethics described in http://biblio.unibe.ch/portale/elibrary/BOP/jemr/ethics.html and that there is no conflict of interest regarding its publication.

### Acknowledgements

The authors acknowledge the University of Costa Rica for financial support provided to Leonela González Vides in her academic training abroad as a fellow under the Academic Mobility program of the Office of International Affairs and External Cooperation.

### Funding sources

No funding was received from funding agencies in the public, commercial, or non-profit sectors.

## Appendix


