# Supplementary material for: Eye Tracking in Optometry: A Systematic Review
Source: J Eye Mov Res. 2023 Aug 16;16(3):10.16910/jemr.16.3.3. doi: 10.16910/jemr.16.3.3 (PMC10725735; doi:10.16910/jemr.16.3.3)
Supplement: Supplementary file 1 [file jemr-16-03-c-SD1-01.pdf]

Appendix 1. Main devices and metrics used in the different areas of optometry

Application area	Devices used	Metrics	Benefits of eye-tracker technology<br><br>Limitations and methodological aspects	References
**Nystagmus**	-Eyelink 1000 (1000 Hz) (n=2)<br><br>-IRIS limbal tracker (100 Hz)<br><br>-Pupil- Labs head-mounted eye tracker (Berlin, Germany)	-Fixations /Fixation stability (n=2)<br><br>-Eye displacement<br><br>-Calibration parameters	- Improved diagnosis<br><br>- Nystagmus waveform characterization<br><br>Small sample size<br><br>Visual function not recorded in some reports<br>No control group<br><br>Statistical analysis: correlation coefficients, coefficients of variation, test-retest variability. These data missing in 3 reports	Thomas et al. (2022)<br><br>Abadi et al. (2021)<br><br>Norouzifard et al. (2020)<br><br>Rosengren et al. (2020a)
**Visual acuity**	-Optics Scanning Laser Ophthalmoscopy- based microstimulation<br><br>-Tracking Scanning Laser Ophthalmoscope (TSLO)<br><br>-Tobii Glasses 1 mobile head-mounted eye tracker (Tobii Technology, Inc., Falls Church, VA) (n=2),<br><br>-Eyelink 2000. (1000 Hz) (SR Research, Mississauga, Ontario, Canada) (n=2)	-Fixations/small fixations (n=4)<br><br>-Eye position<br><br>-Gaze transitions<br><br>-Gaze direction<br><br>-Eye pursuit/ smooth pursuit (n=2)<br><br>-Kinetic visual acuity<br><br>-Dynamic visual acuity<br><br>-Velocity<br><br>-Saccades	-Visual performance and visual acuity assessment<br><br>Small population samples (<10)<br><br>Visual function not recorded in some reports<br>Control group in only 1 report<br><br>Statistical analysis: ANOVA, correlation tests, bivariate correlation coefficients	Domdei et al. (2021)<br><br>Freedman et al. (2019)<br><br>Chen & Yeh (2019)<br><br>Freedman et al. (2018)<br><br>Ağaoğlu (2018)<br><br>Palidis et al. (2017)
**Visual field**	-Glaucoma Module of the Spectralis SD-OCT(Heidelberg Engineering, Heidelberg, Germany)<br><br>-Tobii TX300<br><br>-Eyelink 1000 remote (SR Research, Ontario, Canada) (n=5)<br><br>-Tobii IS-1 eye tracker<br><br>-Tobii X50 eye tracker	-Fixations (n=3)<br><br>-Smooth pursuit/ pursuit eyemovementt (n=4)<br><br>-Gaze tracking/eye gaze (n=2)<br><br>-Saccades (n=3)	-Detection of visual field defects<br><br>-Impact of peripheral vision loss<br><br>-Visual field loss characterized<br><br>-Visual field loss in daily life<br><br>Small population samples (<50)<br><br>Visual function not recorded in some reports<br>Control group in only 1 report<br><br>BCEA formula reported<br><br>Statistical analysis: median, linear-mixed-effects model, ANOVA, correlation coefficients	Liu et al. (2021)<br><br>Chow-Wing-Bom et al. (2020)<br><br>Woutersen et al. (2020)<br><br>Grillini et al. (2018)<br><br>Murray et al. (2018)<br><br>Barraza-Bernal et al. (2017a)<br><br>Barraza-Bernal et al. (2017b)<br><br>Liu et al. (2017)<br><br>Shanidze et al. (2017)
**Amblyopia/ strabismus/vergences**	-Eyelink 1000 remote (SR Research, Ontario, Canada) (500 Hz) (n=9)<br><br>-Tobii EyeX. 60 Hz	-Fixational saccades/ saccades (n=3)<br><br>-Fixational stability/ Average fixation duration/ Number of	- Impact of treatment<br><br>-Visual search performance<br>-Study of fixation stability	Aizenman & Levi (2021)<br><br>Satgunam et al. (2021)<br><br>Murray et al. (2022)

	-iView X, (SensoMotoric Instruments) (60 Hz) (n=2=<br>-ViewPoint EyeTracker system (Arrington Research, Scottsdale, AZ) (350 Hz)<br>-Clinical Eye Tracker system (Version 18.04, Thomson Software Solutions, Hatfield, UK) 780 Hz)<br>-SMI Red250 tracker<br>-SCAN RK-826PCI binocular tracking system (ISCAN, Woburn, MA) (n=2)<br>-Eye-tracker embedded in the stereoscopic virtual reality system EVA (Eye and Vision Analyzer, Davalor Salud, Spain)<br>-Others non-specific: Dual Purkinje image eye tracker	fixations/Time of fixations/Fixations (n=7)<br>-Run count<br>-Eye and gaze position<br>-Eye position (n=7)<br>-Peak velocity<br>-Smooth pursuit<br>-Binocular gaze position<br>-Saccades amplitude<br>-Vergence eye movements (n=2)<br>-Symmetrical disparity vergence<br>-Saccades<br>-Eye movements<br>-Pupil size<br>-Pupil response	-Strabismus diagnosis<br>-Study of ocular deviation<br>-Measurement of heterophoria<br>Small (<50) and large (160>) sample sizes<br>Pre-post evaluation in only 1 report<br>Use of real images<br>Control group used in only 1 report<br>Visual function not recorded in some reports<br>Influence of aging<br>BCEA formula reported<br>Statistical analysis: Levene's and Mauchly's tests, ANOVA, Welch's test, K-S test, paired t-test, Friedman test, Spearman's rank correlation, means, standard deviation, Wilcoxon rank sum test, independent t-test, simple correlation, Shapiro-Wilk-test, U Mann-Whitney test, Pearson correlation, linear regression analysis, one sample t-test	Al-Haddad et al. (2019)<br>Kelly et al. (2019)<br>Tsirlin et al. (2018)<br>Zrinscak et al. (2021)<br>Economides et al. (2021)<br>Mihara et al. (2020)<br>Adams et al. (2017)<br>Kim et al. (2022)<br>Mestre et al. (2021)<br>Gantz & Caspi. (2020)<br>Ramakrishnan & Stevenson (2020)<br>Namaeh et al. (2020)<br>Mestre et al. (2018)<br>Alvarez et al. (2017)<br>Feil et al. (2017)<br>Mestre et al. (2017)
**Technology/visual equipment/virtual and augmented reality/videogames**	-Vive Pro Eye (HTC Corporation) Head Mounted Display with eye-tracking capability<br>-EyeLink 1000 Plus eye tracker (SR Research, Ontario, Canada) (1000 Hz) (n=3)<br>-VOG (EMR-9, NAC Image Technology Inc., Tokyo, Japan)<br>-Tobii Nano eye tracker (Tobii Technology, Sweden) (60 Hz)<br>-Eyecatcher hardware with a Tobii EyeX (Tobii Technology, Stockholm, Sweden) (n=2)<br>-Saccadic Vector Optokinetic Perimetry (SVOP)<br>-Tobii TX300 eye tracker (n=2=<br>-EyeTurn mobile app	-Fixation / Fixed vision trajectory/ fixation stability (n=10)<br>-Saccades /saccades amplitudes/frequency of saccades (n=3)<br>-Microsaccades<br>-Horizontal gaze<br>-Smooth pursuit (n=3)<br>-Calibration<br>-Eye positions (n=2)<br>-Time of reading<br>-Eye deviations in a horizontal direction<br>-Gaze angles<br>-Head translation/head rotation	-Visual performance<br>-New methods of calibration<br>-Validation of new eye-tracker<br>-Development of virtual reality<br>-Smartphone application<br>-Improved image quality in OCT-A<br>Small (<10) and large (162>) sample sizes<br>Control group included in some studies<br>Pretest-postest in only 1 report<br>Visual function is not registered in some papers<br>Statistical analysis: paired t-test, Kruskal-Wallis test, Mann-Whitney test, linear regression, simple correlation, Pearson`s correlation test, Bonferroni test, Shapiro-Wilk test, Wilcoxon rank sum test<br>Limitations reported: sample size	Fujimoto et al. (2022)<br>Essig et al. (2021)<br>Love et al. (2021)<br>Hirota et al. (2021)<br>Mao et al. (2021)<br>Rosengren et al. (2020b)<br>Jones (2020)<br>Tatham et al. (2020)<br>Kim et al. (2019)<br>Jones et al. (2019)<br>Esfahlani et al. (2019)<br>Pundlik et al. (2019)<br>Hirasawa et al. (2018)<br>Cheong et al. (2018)<br>Albert et al. (2017)<br>Chopra et al. (2017)<br>Lauermann et al. (2017)<br>Murray et al. (2017)

	-Tobii glass II (TobiiTechnology, Stockholm, Sweden) 50 Hz<br><br>-SMI eye tracker (250 Hz)<br><br>-OCT-system (AngioVue, RTVue XR Avanti SD-OCT, Optovue,Fremont, CA, USA) (n=2)<br><br>-Tobii IS-1 model (Tobii Technology, Stockholm, Sweden<br><br>-Others non specific: Own wearable Display Prototype			
**Eye movements/saccades/smooth pursuit/ fixations/microsaccades/processing speed/ gaze stability**	-Adaptive Optics Scanning Laser Ophthalmoscope (AOSLO) 960 Hz (n=2)<br><br>-Eye-movement Real-time Integrated System (EyeRIS)<br><br>-Dual Purkinje Image (DPI)method, a generation 6 analog DPI eye tracker (Fourward Technologies). (n=2)<br><br>-EyeLink1000 (SR Research, Ottawa, Ontario, Canada) (1000Hz) (n=8)<br><br>-iView XTM High Speed 1250 IT<br><br>-Mobile eye tracker (SMI BeGaze; SensoMotoric Instruments)<br><br>-Sensor motoric instruments eye tracking glasses (SMI ETG) 120 Hz<br><br>- Tobii T60XL eye tracker (Tobii Corporation, Sweden)<br><br>- Infrared eye tracker (USB-220, Arrington Research, Scottsdale, AZ)<br><br>- Eye Tracking Glasses 2.0 (ETG 2.0; SensoMotoric Instruments, Teltow, Germany) 60 Hz<br><br>- Monocular eye-tracking system, EyeSeeCam, 220 Hz (ESC; EyeSeeTech GmbH, Furstenfeldbruck, Germany)<br><br>- Others non-specific:<br><br>1.A built high-resolution binocular eye tracker using two USB3 infrared monochrome cameras having 640x480 pixel	-Fixations/fixation duration /reaction time to fixation (n=10)<br><br>-Saccades /pursuit saccades/saccades amplitude (n=7)<br><br>-Eye position/gaze position stability (n=2)<br><br>-Microsaccades (n=6)<br><br>-Monocular eye movements<br><br>-Binocular eye movements<br><br>-Postural sway<br><br>-Head movements<br><br>-Vertical and horizontal rotations of the eye	Compare fixational eye movements<br><br>Study how microsaccades work<br><br>Study patterns of fixations<br><br>Study eye movements in children<br><br>Study human saccades<br><br>Integrating an eye-tracker into an eye-implant for blind people<br><br>Characterize gaze stability<br><br>Small (<20) and large (241>) sample sizes<br><br>Sample size: arbitrary size in only 1 report<br><br>Pilot study in only 1 report<br><br>Visual function is not registered in some papers<br><br>Eye-tracking paradigm was reported.<br><br>Statistical analysis: correlation test, mean, median, standard deviation, Shapiro-Wilk test, Welch's test, ANOVA	Bowers et al. (2021)<br><br>Intoy et al. (2021)<br><br>Nanjappa & McPeek. (2021)<br><br>Belyaev et al. (2020)<br><br>Raveendran et al. (2020)<br><br>Poletti et al. (2020)<br><br>Chaudhary et al. (2020)<br><br>Ivanchenko et al. (2019)<br><br>Pel et al. (2019)<br><br>Kelly et al. (2019)<br><br>Kwon et al. (2019)<br><br>Badler et al. (2019)<br><br>González et al. (2019)<br><br>Goettker et al.(2019)<br><br>Fang et al. (2018)<br><br>Caspi et al. (2018)<br><br>Ratnam et al. (2018)<br><br>Seemiller et al. (2018)<br><br>Sheynikhovich et al. (2018)<br><br>Amit et al. (2017)<br><br>Vater et al. (2017)

	resolution, and sampling images at 400 Hz (The Imaging Source, Model DMK33UX174)<br>2. Binocular Dual Purkinje Image eye tracker<br>Revolving Field Monitor, a specially designed eye-coil apparatus			
**Ocular pathology/low vision/glaucoma/stardgart disease/AMD/diabetic macular edema**	-Retinal image-based eye tracker, the TSLO (C. Light Technologies, Inc., Berkeley, CA)<br>- Eyelink 1000. (SR Research, Ontario, Canada) (n=4)<br>- Tobii Glasses Pro 2<br>- Iscan ETL 100Hz (MA, USA)<br>- Stereoscopic eye tracking system with two USB 3.0 cameras and two infrared light. 300 Hz<br>- Tobii T60XL (Tobii Corporation, Danderyd, Sweden<br>- Tobii TX300eye-tracker (Tobii Technology, Danderyd, Sweden) (n=3)<br>- Spectralis self-acting eye-tracking (eye tracker)<br>- iView X™ video-based eye tracker<br>- External infrared eye-tracking camera (Arrington Research,Inc., Scottsdale, AZ, USA<br>- video-based eye-tracker (Series 2020; El-Mar, Inc., Toronto, ON, Canada) 120 Hz	-Fixations/fixation time/reaction time to fixation/fixation duration/ spread of fixation locations/monocular and binocular fixations (n=10)<br>-Saccades/saccade amplitude (n=9)<br>-Microsaccades<br>-Blinks<br>-Drifts<br>-Smooth pursuit<br>-Gaze position (n=3)<br>-eye movements (n=2)<br>-Horizontal and vertical variance	Evaluate fixational eye movements in people with ocular diseases.<br>Determine eye movement differences.<br>Visual functions assessment<br>Type of study: prospective experimental cohort study (n=1), longitudinal study (n=1), case control study (n=1),<br>cross-sectional study in only 1 report<br>Small (<50) and large (187>) sample sizes<br>Control groups were used in some papers.<br>Some do not describe population .<br>Visual function is registered.<br>BCEA formula was calculated.<br>Statistical analysis: Mann-Whitney U test, two-sample t test, Spearman`s correlation, chi-square test, coefficient of variation, test-retest variability, ANOVA, Shapiro-Wilk test	Leonard et al. (2021)<br>Ballae Ganeshrao et al. (2021)<br>Garric et al. (2021)<br>Giacomelli et al. (2020)<br>Senger et al. (2020)<br>Titchener et al. (2020)<br>Barsingerhorn et al. (2019)<br>Lee et al. (2019)<br>Kooiker et al. (2019)<br>Asfaw et al. (2018)<br>Laude et al. (2018)<br>Gao & Sabel. (2017)<br>Abadia et al. (2017)<br>Lee et al. (2017)<br>Jakobsen et al. (2017)<br>Shivdasani et al. (2017)<br>Tarita-Nistor et al. (2017)<br>Alberti & Bex. (2017)
**Assessment/diagnosis/visual function/Rehabilitation/training**	-Tobii 4c eye tracker. 90 Hz (n=5)<br>-Tobii-X2-30 eye tracker<br>-Eye tracker inside Saccadic Vector Optokinetic Perimetry (SVOP)<br>-Tobii IS-1 (TobiiTechnology, Stockholm, Sweden) 40-H<br>- Mobile Eye Tracker (Mobile EBT®)	-Eye movements<br>-Eye velocity<br>-Pupil size (n=3)<br>-Horizontal gaze/gaze data (n=4)<br>-Blink rate (n=3)<br>-Eye position	Colour vision assessment<br>Training in visual rehabilitation<br>Quantify saccades, smooth pursuit and contrast sensitivity<br>Visual assessment in infants and people with disabilities.<br>Nystagmus treatment<br>Training visual abilities<br>Automatic visual impairment detection system	Taore et al. (2022)<br>Awada et al. (2022)<br>Wilhelmsen et al. (2021)<br>Brodsky & Good. (2021)<br>Xie et al. (2021)<br>Mooney et al. (2021)<br>Perperidis et al. (2021)<br>Chatard et al. (2019)

	- Generation 6 Dual Purkinje Image (DPI) eye tracker (Fourward Technologies)<br><br>- Eye-link 1000+ eye-tracker (SR Research) (n=2)<br><br>- Tobii Pro X3-120 (Eye Tracker; Tobii AB Inc., Danderyd, Sweden)<br><br>- Tobii X2–60 eye tracker<br><br>- SMI eye 94 tracker (SensoMotoric Instruments GmbH, Teltow, 95 Germany) (n=2)<br><br>- HMD-ET. 240 Hz<br><br>- Tobii PRO TX-300 (n=3)<br><br>- Comprehensive Oculometric Behavioral Response Assessment (COBRA)<br><br>- Nidek MP-1 microperimeter (Navis, Nidek Technologies, Italy)	-Smooth pursuit/pursuit latency/ pursuit (n=3) acceleration (n=2)<br><br>-Saccades/ saccades amplitude/ saccades frequency (n=5)<br><br>-Microsaccades<br><br>-Monocular central fixation/fixations (n=5)<br><br>- Time to looking at targets or distractors<br><br>- Eye movements (n=3)<br><br>- Reading<br><br>- Fixations	Type of study: prospective study in only 1 report<br><br>Small (<60) and large (187>) sample sizes<br><br>Some do not describe population<br><br>Visual function is registered.<br><br>Statistical analysis: ANOVA, independent test, Kruskal-Wallis test, paired t-test, Pearson`s correlation coefficient, Spearman`s correlation, paired Student`s t- test, Mann-Whitney test	Shelchkova & Poletti. (2020)<br><br>Ramesh et al. (2020)<br><br>Weaterton et al. (2020)<br><br>Wan et al. (2020)<br><br>Wang et al. (2020)<br><br>Tatiyosyan et al. (2020)<br><br>Hotta et al. (2019)<br><br>Axelsson et al. (2019)<br><br>Shelchkova et al. (2019)<br><br>Wen et al. (2018)<br><br>Mooney et al. (2018)<br><br>Ryu & Wallraven (2018)<br><br>Liston & Stone. (2017)<br><br>Liu et al. (2017)<br><br>Schönbach et al. (2017)<br><br>Yow et al. (2017)
**Surgery**	-Mobile T2 (Suricog, France) 300 Hz<br><br>- Motion-tracking software (PHACOTRACKING,Guildford, United Kingdom)<br><br>-Others non-specific: Custom-built eye tracker	-Fixation /fixation light tracking<br><br>- Bell's reflex<br><br>- Saccades<br><br>-Oscillations<br><br>- Nociceptive reflex movement<br><br>- Nystagmus	Evaluate differences in eye movements<br><br>Small sample size (<10)<br><br>Visual function is not registered in some papers<br><br>BCEA formula was calculated.<br><br>Statistical analysis: descriptive statistic	Perrin-Fievez et al. (2018)<br><br>Reinstein et al. (2018)<br><br>Coletta et al. (2018)
**Refractive error**	- Eyelink 1000 Plus, SR Research, Ontario, Canada) 500 Hz (n=2)	-Horizontal and vertical eye movements<br><br>-Centre of rotation of the eye<br><br>- Eye position<br><br>- Eye velocity<br><br>-Saccades<br><br>-Fixation stability	Study the eyeball rotation in refractive error.<br><br>Study refractive error<br><br>Small sample size (<59)<br><br>Statistical analysis: ANOVA<br><br>Limitation reported: calibration, reflections on surface lenses.	Ohlendorf et al. (2022)<br><br>Doustkouhi et al. (2020)
**Reading**	-MAIA microperimetry	-Fixation/ fixation duration / first fixation	Analyze the effect of text presentation, color, and size in eye movements.	Bowman et al. (2021)

	- Eyelink II (SR Research, Mississauga, Ontario, Canada) 500 Hz<br><br>- SMI eye tracker (RED 250 m, SensoMotoric)<br><br>- Tobii TX300 eye tracker (Tobii Tecnoloy Danderyd, Sweeden) 300 Hz (n=4)<br><br>- Mobile T2 (SuriCog, France) 300 Hz<br><br>- Tobii XL120, 120Hz	duration/fixation stability (n=7)<br><br>-Gaze duration<br><br>-Selection regression-path duration (n=2)<br><br>- Reading time/ reading speed (n=3)<br><br>- Saccades/number and amplitude of saccades (n=4)<br><br>- Blinks	Measure reading rates<br><br>Small (<50) and large (120>) sample sizes<br><br>Sample size calculated with Cochran`s formula in only 1 report<br><br>Control group was used in some papers.<br><br>Visual function is not registered in some papers<br><br>Statistical analysis: Shapiro-Wilk-test, Wilcoxon rank sum-test, ANOVA, Mann-Whitney test, chi-square<br><br>Limitations reported: The use of glasses and sample size.	Hyona et al. (2020)<br><br>Wertli et al. (2020)<br><br>Fadzil et al. (2019)<br><br>Murata et al. (2017)<br><br>Ridder et al. (2017)<br><br>Ryu & Wallraven. (2017)<br><br>Vinuela-Navarro et al. (2017)
**Sport vision/ locomotion**	-EyeLink 1000 (SR Research Ltd., ON,Canada)	-Saccades<br><br>-Eye response time	Effects of visuomotor training<br><br>Small sample size (<56)<br><br>Visual function is not registered in some papers<br><br>Statistical análisis: descriptive statistic (mean, standard deviation), comparative statistic	Ju et al. (2018)
**Oculomotor deficits/ oculomotor responses**	-Device for an Integral Visual Examination (DIVE) 60 Hz<br><br>- C-ETD (Chronos Inc., Berlin)	-Fixations<br><br>-Saccades<br><br>-Binocular tracking<br><br>- Horizontal and vertical eye movements<br><br>- Torsional eye movements	Study oculomotor behavior in children and young people<br><br>Small sample size (<29)<br><br>BCEA formula was calculated.<br><br>Statistical analysis: mean, standard deviation, Kruskal-Wallis test, MANOVA, Shapiro-Wilks test	Pueyo et al. (2020)<br><br>Wibble et al. (2020)

Appendix 2. Relevant information of each included study

Year	Main Objective	Subjects	Device Characteristics	Metrics	Method aspects	Reference
2022	Get more knowledge about their functional vision as a fundament for educating pupils with albinism	3 subjects (10-12 years old)	Tobii Eye Tracker 4 C	Pupil size<br><br>Horizontal gaze	Environment:<br><br>Iluminating and reflects were controlled.<br><br>Stimuli: Horizontal smooth pursuit task.<br><br>Distance:60 cm<br><br>E-T benefits: could reveal if any child could really keep a fixed gaze position during reading as this often results in abnormal head postures and neck problems	Wilhelmsen et al.
2022	Develop a simple objective test of functional colour vision based on eye movements made in response to moving patterns	34 participants (17 females, 17 male, 17–65 years), of which 23 were normal trichromat controls, 9 were deuteranopes and 2 were protanopes	Tobii 4c Eye tracker. 90 Hz	Eye movements<br><br>Eye velocity<br><br>Pupil diameter	Laptop 1920x1080 pixels, at 40 cm<br><br>Stimuli: DEM test<br><br>E-T benefits: Advances in software-based eye tracking running on many devices equipped with front-facing cameras their test could become a simple, reliable, automated colour vision assessment that could be downloaded for use by clinician.	Taore et al.
2022	Assess the effects of monocular, binocular, and dichoptic viewing on FEMs and eye alignment in patients with and without fusion maldevelopment nystagmus (FMN)	34 patients with amblyopia and 7 healthy controls<br><br>Subjects were randomly selected<br><br>Control group	EyeLink 1000<br><br>Chin rest	Fixational saccades<br><br>Fixation stability<br><br>Intersacadic microsaccades	Equipment: Monitor of 1280 x 800 pixels<br><br>Binocular horizontal and vertical eye positions were measured.<br><br>Dark room<br><br>Stimuli: A white circular target was used.	Murray et al.

					Calibration and validation of each eye was done.<br><br>Statistical analysis: MatLab was use to analyze eye positions, with Engbert and Kleigl algortih,<br><br>SPSS to statistical analysis, Test Kolmogorov-Smirnov, Levene`s test and Mauchly^s tests<br><br>ANOVA.<br><br>E-T benefits: FEM abnormalities modulate with different viewing conditions as used in various amblyopia	
2022	Establish a quantitative evaluation and comparison of fixation stability, as measured by an eye tracker, using image-based areas determined by the bivariate contour ellipse area (BCEA), kernel density estimation (KDE), and Scanpath methods.	45 and 20 participants with abnormal and normal phoria (29 women, mean age 21-5 +/- 1.9 years)	Clinical EyeTracker system (Version 18.04, Thomson Software Solutions, Hatfield, UK) 70 Hz	Fixation stability<br><br>Binocular gaze position	Data analysys: Paired-sample t-test in SPSS<br><br>Wilconxon text	Kim et al.
2022	Conduct preliminary eye and head mount display (HMD) movement measurements to collect primary data to create a Spatio-temporal virtual reality(VR) navigation system	11 university students (aged 21-22 years)	Vive Pro Eye (HTC Corporation),<br><br>and Head Mounted Display with eye-tracking capability	Fixation<br><br>Fixed vision trajectory<br><br>microsaccades	Stimuli: fixed vision, random bouncing vision, and liner motion chasing.	Fujimoto et al.
2022	Study if the training with optic flow stimuli promotes recovery in cortical blindness	-	-	-	E-T benefits: permits assessment and training in cortical blindness.	Awada et al.
2022	Study a voluntary Flutter Presenting During Ophthalmoscopy in an unusual case	1 male with autism<br><br>20 years old	Eyelink 1000 (SR Research, Ottawa, ON, Canada) 1000 Hz<br><br>A chinrest was used.	Fixations<br><br>Monocular fixations	E-T benefits: permits register of voluntary flutter to diagnosis between nystagmus or other conditions.<br><br>Stimuli: fixation in five points, monocular fixation in primary position, binocular	Thomas et al.

					convergence, seven minutes central fixations.	
2021	Study Fast and nonuniform dynamics of perisaccadic vision in the central fovea	8 subjects (6 females, age range 23-33 years)	Eye-movement Real-time Integrated System (EyeRIS)<br><br>Dual Purkinje Image (DPI)method, a generation 6 analog DPI eye tracker (Fourward Technologies).	Microsaccades<br><br>Saccades	Two-tailed nonparametric bootstrap test<br><br>Head-rest<br><br>Stimuli: 30 gray dots randomly distributed within 2º<br><br>Based-event recorded by EyeRIS	Intoy et al.
2021	Determine whether fixational stability can be used as an objective marker for the recovery of visual function in amblyopia	5 children with amblyopia (mean age 8.2 years)<br><br>5 normally sighted children	Eyelink II 500 Hz	Fixational Saccades	E-T benefits: permits measure treatment in amblyopia.<br><br>Stimuli: 1º colorful smiley face for 20 seconds.<br><br>Pre and post evaluation	Aizenman, A. & Levi, D.
2021	Present a new computational approach to analyze nystagmus waveforms	5 adults, 1 male (age range 22-46 years)	IRIS limbal tracker.(Skalar Medical, Delft, The 1 Netherlands).<br><br>100 Hz. Head-mounted<br><br>Video-based infra-red pupil tracking eye tracker. 400 Hz. (Chronos: Skalar Medical, Delft, The Netherlands)	Fixation stability<br><br>Amplitude saccades		Abadi et al.
2021	Compare saccadic eye movements between high-tension glaucoma (HTG), normal-tension glaucoma (NTG), and primary angle-closure glaucoma (PACG)	52 subjects<br><br>15 HTG, 14 PACG, 8 NTG<br><br>15 normal controls	Eyelink 1000	Saccades<br><br>Prosaccades	Gap paradigm<br><br>3 target eccentricities<br><br>Latency, average and peak velocity of prosaccades.<br><br>E-T benefits: Determine eye movements differences between types of glaucoma	Ballae et al.

2021	Compare and contrast fixational eye movements (FEM) during *active* tasks–those that contain temporal variation and require subject input—and *passive* tasks, where the subject is simply instructed to maintain fixation on a target	8 healthy subjects (3 male, ages range 23 to 53 years)	Adaptive Optics Scanning Laser Ophthalmoscope (AOSLO) 960 Hz	Fixations<br><br>Microsaccades	Stimuli:fixation targets (Maltese cross, disk, concentric circles, Vernier and tumbling-E letter)	Bowers et al.
2021	Investigate the conjoint effects of color mode and luminance contrast on visual fatigue and subjective preference when using electronic devices under low screen luminance and low ambient illumination at night	60 subjects (25 female)	Tobii-X2-30 eye tracke	Pupil data<br><br>Blink rate	Stimuli: middle contrast texts, 21 pages with 400 words per page.<br><br>Statistical analysis. MANOVA<br><br>E-T benefits: allowed get some guidelines for the design of interaction interface of the electronic devices in a specific illumination condition.	Xie et al.
2021	Compare rapid serial visual presentation (RSVP) and horizontal scrolling text presentation (scrolling) on reading rate and reading acuity in CVL observers and normally-sighted controls with simulated CVL	11 subjects bilateral CVL<br><br>16 controls	MAIA microperimetry<br><br>EyeLink 1000 Pluseye tracker (SR Research, Ontario, Canada) with fixed sampling rate of 1000 Hz	Fixation	Stimuli: rapid serial visual presentation texts, a green fixation dot 4º above the text<br><br>E-T benefits permits to analyze the effect of the text presentation and font size on reading.	Bowman et al.
2021	Test the vision in subjects with visual impairment	Visual impairment people	-	-		Brodsky & Good.
2021	Assess to what extent the specific cellular organization of the foveola of an individual is reflected in visual sensitivity and if sensitivity peaks at the preferred retinal locus of fixation (PRL)	4 human participants (one female; ages 29, 32, 42, and 42 years)	Optics Scanning Laser Ophthalmoscopy- based microstimulation	Small fixations	Stimuli: small-spot, cone targeted visual stimuli<br><br>allowed to develop a model of visual sensitivity in the foveola, with distance from the PRL (eccentricity), cone density, and OS length as parameters	Domdei et al.
2021	Develop and validate an automated contrast sensitivity (CS) test using a live- detection of optokinetic nystagmus (OKN)and an adaptive psychometric procedure	15 healthy participants four male, (mean age 24.7 +/- 3)	EyeLink 1000 Pluseye tracker (SR Research, Ontario, Canada) with fixed sampling rate of 1000 Hz	Saccade<br><br>Horizontal gaze<br><br>Blink detection	Stimuli: vertically oriented square-wave grating drifting over the horizontal plane with a constant velocity<br><br>Statistical analysis: Matlab to analyze data	Essig et al.

					E-T benefits: Possibility to use OKN to assess visual performance, visual acuity, visual field	
2021	Investigate saccadic movements in subjects with eccentric fixation due to a deep central scotoma in Stargardt disease (STGD)	10 patients with STGD and 10 healthy subjects (control group)	Tobii Glasses Pro 2	Saccadic eye movements<br><br>Fixations	Chin rest<br><br>Stimuli: spot of 1° angular size which started in the center or in four different positions<br><br>Statistical analysis: two-sample t-test, with equal or unequal variance as appropriate. A paired t-test was used The correlation pattern among saccadic times and psychophysical variables was analyzed using Spearman correlation.	Giacomelli et al.
2021	Estimate the impact of glaucoma on computer use and assess specific adaptations of the graphical interface to this form of visual impairment	49 subjects: 16 patients with primary open-angle glaucoma (mean 62.7 ± 5.6 years of age), 17 age-matched participants (mean 59.1 ± 8.3 years of age), 16 young control participants (mean 23.3 ± 2.1 years of age)	-	Oculomotor behavior	Prospective experimental cohort study<br><br>Stimuli: ecological computer scenes with 3 enhancement levels (low, medium, and high), determined by gradual modulation of contrast, luminance, and color<br><br>To improve the interface features to improve visual comfort.	Garric et al.
2021	Analyze the amplitude, direction, and rate of small saccades as a function of vergence demand when testing the near point of convergence.	11 young adults (mean age ± SD: 25.4 ± 2.2 years)	EyeLink 1000 Plus (SR Research)	Eye positions<br><br>Saccades amplitude<br><br>Number of saccades per second	Chin rest<br><br>Statistical analysis: MatLab and SPSS<br><br>Parametric test, Shapiro-Wilk test, Spearman`s rank order, Mann-Whitney test and Kruskal-Wallis test were used.<br><br>Stimuli: fixation cross which moved along the midline to elicit symmetrical conver-<br><br>gence and divergence movements	Mestre et al.
2021	Explore the different eye movement behaviors of people with several types of artificial Visual Field Defects	38 participants with normal vision were recruited to watch a group of videos monocularly	Tobii Nano eye tracker (Tobii Technology, Sweden) 60 Hz	Smooth pursuit<br><br>Fixations	Stimuli: 41 videos with duration less than 2 minutes.	Mao et al.

	(VFDs), such as hemianopia, altitudinal VFDs, and tunnel vision				Statistical analysis: Non parametric test, Kruskal-Wallis, Mann-Whitney test were used.<br><br>It was proved in normal people, is unclear if the test works in VFD patients	
2021	Present a novel, properly tested and evaluated eye-tracking based method for manifest strabismus diagnosis is presented.	81 patients, 41 with a diagnosis of strabismus	Tobii EyeX. 60 Hz<br><br>Dual Purkinje Image eye-tracker	Eye and gaze positions	Distance: 60 cm<br><br>Stimuli: strabiscope hardware<br><br>Statistical analysis: SPSS, Kolmogorov-Smirnov	Zrinšćak et al.
2021	Describe a novel approach to grading visual health based on eye movements and evidence from gaze-based tracking behaviors.	14 children (age range 3 to 18 years)	Tobii 4C eye tracker (50–95 cm operating distance; 90 Hz sampling rate)	Gaze data<br><br>Saccades<br><br>Pursuits	Distance: 62 cm<br><br>Stimuli: Visual Ladder program, 5 tasks.<br><br>Statistical analysis: to detect broad asymmetries in saccade amplitude specifically, independent-tests<br><br>E-T benefits: Quantify saccades, smooth pursuits,and contrast sensitivity in children of a wide variety of ages and communicative ability,	Mooney et al.
2021	Compare visual field results of the COMPASS fundus perimeter (CMP) and the Humphrey Field Analyzer (HFA) in the same eyes.	124 eyes of 79 patients with glaucoma	Glaucoma Module of the Spectralis SD-OCT(Heidelberg Engineering, Heidelberg, Germany)	-		Liu et al.
2021	Quantify reductions in calibration accuracy relative to fixation eccentricity and suggest a robotic calibration and validation tool.	8 subjects with central field loss (75.1 +/- 12.1) and 7 controls (72.4 /- 5.7)	Eyelink 1000<br><br>EyeRobot	Fixations	E-T benefits: New methods of calibration in CFL	Love et al.
2021	Evaluate fixational eye movements (FEMs) with high spatial and temporal resolution following concussion.	99 adolescents and young adults (13 to 27 years old)	Retinal image-based eye tracker, the TSLO (C. Light Technologies, Inc., Berkeley, CA)	Fixations<br><br>Saccades<br><br>Drifts	Stimuli: three fixation tasks<br><br>Statistical analysis: t-test or Mann–Whitney U, Kolmogorov-Smirnov	Leonard et al.

2021	Investigate visual search performance in this cohort with real world images	23 patients (mean age: 10 ± 0.6 years) and 13 controls (10 ± 0.9 years)	Eyelink1000	Saccades count<br><br>Run count (number of repeating viewing)<br><br>Average fixation duration	Stimuli: A real world image was displayed on a computer screen along with a search target	Satgunam et al.
2021	Determine how the position of the centre of rotation of the eyeball is related to axial length and refractive error when horizontal and vertical eye movements are performed.	59 subjects (32 females, age range 36.3 +/- 9.1 years)	Custom-built eye tracker	Horizontal and vertical eye movements<br><br>Centre of rotation of the eye	Statistical analysis: ANOVA	Ohlendorf et al.
2021	Development of a Simple Test of the Slow-To-See Phenomenon in Children with Infantile Nystagmus Syndrome	Patients with nystagmus, diagnosis of learning disability	Eye-link 1000+ eye-tracker (SR Research)	Time looking at targets and distractors	Stimuli: find their own mother or a target face from a selection of faces displayed at once on a tablet and then press the target as fast as possible<br><br>Statistical analysis: SPSS, Spearman`s rank coefficient.<br><br>E-T benefits: Use of e-t in clinical trials of nystagmus treatment.	Weaterton et al.
2021	Compare the ocular deviation in patients with intermittent exotropia under conditions of monocular versus binocular occlusion	18 Subjects (11 females, range 8-60 years)	Two video-based eye trackers (iView X; Senso-Motoric Instruments, Teltow, Germany), sampling at 60 Hz	Right and left eye positions	Task: Fixate a target.<br><br>Stimuli: spot of light 0.5º in diameter.<br><br>Statistical analysis: paired t-test	Economides et al.
2021	Accurately record the movements of a hand-held target together with the smooth pursuit eye movements (SPEMs) elicited with video-oculography (VOG) combined with deep learning-based object detection using a single-shot multibox detector (SSD)	11 healthy subjects (21.3 +/- 0.9 years)	VOG (EMR-9, NAC Image Technology Inc., Tokyo, Japan	Smooth pursuit eye movements	Stimuli: rabbit-like character, size 10 x 10 cm.<br><br>Statistical analysis: paired t-test. SPSS program to determine significance differences.	Hirota et al.

2021	Study microsaccades and attention in a high-acuity visual alignment task	11 subjects (six female, age range 25-30 years)	EyeLink1000 (SR Research, Ottawa, Ontario, Canada) at 1000Hz.	Monocular eye movements<br><br>Fixations<br><br>microsaccades	Distance: 120 cm<br><br>Task: 2 experiments, 120 trials ~40 minutes, fixate a target without blink.<br><br>Detect a letter in a RSVP.<br><br>E-T benefits: how to microsaccades works in fine acuity tasks	Nanjappa & McPeek.
2020	Determine threshold visual field sensitivities in normal subjects performing saccadic vector optokinetic perimetry (SVOP), a new eye tracking perimeter	113 healthy patients<br><br>(Mean age 65.9 +/-10.1)	Saccadic Vector Optokinetic Perimetry (SVOP)	-	Comparative study<br><br>Statistical analysis: Bland-Altman plots, relationships were examined by pointwise linear regression.	Tatham. et al.
2020	Generate a Real-Time Eye Tracking Method for Detecting Optokinetic Nystagmus	6 healthy subjects	Pupil- Labs head-mounted eye tracker (Berlin, Germany)	Eye displacement	Distance: 1.5 meters<br><br>E-T benefits. Detect optokinetic nystagmus	Norouzifard et al.
2020	Assess oculomotor behavior in children adopted from Eastern Europe, who are at high risk of maternal alcohol consumption	29 adoptees and 29 age-matched controls	Device for an Integral Visual Examination (DIVE) 60 Hz	Fixations<br><br>Saccades	Stimuli: High contrast cartoon on the centre of the screen, short fixational tasks.<br><br>Fixations and saccades were identified with a dispersion-based algorithm.<br><br>Fixation stability calculated with BCEA<br><br>Statistical analysis: mean, SD, ranges. Shapiro-Wilk test, Kruskal- Wallis test to compare, student´s t-test<br><br>E-T benefits: allows better assessment of oculomotor skills.	Pueyo et al.
2020	Study the effect of refractive error on optokinetic nystagmus	Experiment 1. 20 participants (20-35 years old)<br><br>Experiment 2. 25 participants (19-51 years old)	Eyelink 1000 Plus (SR Research, Ontario, Canada) 500 Hz	Saccades	Distance: 1m<br><br>Stimuli: generated in Statistical analysis: Matlab, spatial filtered-two dimensional random noise patterns.<br><br>Limitations: an eye-tracker that could measure EM independent from calibration	Doustkouhi et al.

					and the reflections from surface lenses is needed.	
2020	Develop an OKN-based virtual diagnosis tool to estimate contrast sensitivity automatically without the active cooperation of the patient as well as the practitioner within 3.5 minutes.	12 healthy subjects with normal or corrected to normal vision (mean age 25.2 +/-1.7)	SMI eye 94 tracker (SensoMotoric Instruments GmbH, Teltow, 95 Germany)	Eye movements	Stimuli: fixation point target and motion sequence<br><br>Statistical analysis:<br><br>Matlab<br><br>E-T benefits: good tool to screen visual function in patients with difficulties.	Tatiyosyan et al.
2020	Examine whether assessing the visual functioning of the "intact" ipsilesional visual field can be useful to understand difficulties experienced by patients with visual field defects	18 patients (15 males,) with visual field defects owing to postchiasmatic brain lesions.<br><br>18 control subjects	Eyelink 1000 remote (SR Research, Ontario, Canada)	Gaze tracking	Stimuli: UFOV test<br><br>Statistical analysis: Matlab, mixed-design ANOVA.<br><br>e-t: stimuli UFOV is good for evaluating visual functioning.	Woutersen et al.
2020	Analyze detailed contrast sensitivity function measured with a nonverbal procedure called "Gradiate"	60 subjects (34 females, age range 11 to 74 years)	Tobii 4C eye tracker (operating distance of 50–95cm; sampling rate of 90 Hz)	Smooth tracking	Stimuli: Five targets were presented to the observer simultaneously in each Gradiate trial. Python was used to generate stimuli and extract data<br><br>Limitations: calibration of e-t	Mooney et al.
2020	Observe the change in horizontal smooth pursuit in patients with intermittent exotropia before and after strabismus surgery.	9 patients (mean age, 22.2 ± 13.9 years)	ViewPoint EyeTracker system (Arrington Research, Scottsdale, AZ) at a sampling rate of 350 Hz	Velocity and amplitude of Smooth pursuit<br><br>Eye position	Head positioner was used.<br><br>Statistical analysis: SPSS, Friedman test, Spearman`s rank correlation	Mihara et al.
2020	Study accuracy and precision of small saccades	11 subjects (5 emmetropic, 3 females)	Dual Purkinje imaging for high-resolution eye-tracking	Gaze localization<br><br>Saccades	Distance: 126 cm.<br><br>Head rest	Poletti et al.

2020	Evaluating the impact of asymmetric peripheral vision loss on everyday function	20 healthy adults (mean age 26.2 years) with normal vision	FOVE0 Eye-Tracking VR headset (FOVE Inc., San Mateo, CA, United States) 70 Hz	Eye movements<br><br>Head movements	Stimuli: Search environments consisted of 15 household rooms<br><br>Statistical analysis: Linear-Mixed-Effects model. Matlab	Chow-Wing et al.
2020	Investigate visual fixations and postural sway in response to increasingly complex visual environments in healthy adults and adults with motion sensitivity	20 healthy adults<br><br>20 adults with motion sensitivity	Mobile eye tracker (SMI BeGaze; SensoMotoric Instruments)<br><br>Sensor motoric instruments eye tracking glasses (SMI ETG) 120 Hz	Visual fixations<br><br>Postural sway<br><br>Body kinematics	Stimuli: Python, 6 tasks, each lasting 70 seconds<br><br>Sample size: arbitrary size.<br><br>Statistical analysis: descriptive statistics, Kolmogorov-Smirnov test.	Chaudhary et al.
2020	Establish normative data for objective measures of disparity vergence and saccades in children using an objective binocular eye movement tracking system	118 subjects (age range 9 -17 years)	SCAN RK-826PCI binocular tracking system (ISCAN, Woburn, MA)	Symmetrical disparity vergence<br><br>Saccades	Stimuli: vertically oriented visual stimulus target<br><br>Data analysis: Matlab, SPSS, Shapiro-Wilk test, descriptive statistics	Namaeh et al.
2020	Propose a signal quality metric for nystagmus waveforms, the normalized segment error (NSE)	3 subjects with nystagmus	EyeLink 1000Plus	Calibration<br><br>Fixations<br><br>Smooth pursuit<br><br>Saccades	E-T benefit: characterization and comparison of nystagmus waveform patterns	Rosengren et al.
2020	Investigate oculomotor behavior in response to dynamic stimuli in retinal implant recipients	3 suprachoroidal retinal implant recipients	Head-mounted eye tracker (Arrington Research,Scottsdale, AZ, USA) 60 Hz	Smooth pursuit		Titchener et al.
2020	To compare the impact of unilateral versus bilateral Age-related Macular Degeneration (AMD) on saccadic movements, and to show the effect of visual search training on these eye movement performances in AMD subjects	13 elderly unilateral AMD (mean age 74.6 +/- 1.6 years)<br><br>15 elderly bilateral AMD (mean age 74.2 +/- 1.2 years)<br><br>15 healthy age-matched control (mean age: 70.9 +/- 1.3 years)	Mobile Eye Tracker (Mobile EBT®)	Horizontal saccadic	Dark room<br><br>Head rest<br><br>Data: time, number of omissions and error in saccades.<br><br>Statistical analysis: Kruskal-Wallis and posthoc tests.	Chatard et al.

2020	Synchronization of a Removable Optical Element with an Eye Tracker for Heterophoria Measurement	30 normal sighted subjects (mean age 24.50 +/- 2.20)	SMI Red250 tracker	Eye position	Statistical analysis: Pearson correlation, Anderson-Darling normality test. Matlab	Gantz & Caspi
2020	Study the dependence of the correlation of time spent in fixations on the degree of distortion of texts when reading.	-	iView XTM High Speed 1250 IT	Fixations<br><br>Saccades		Belyaev et al.
2020	Explore how visual and vestibular acceleration affect roll-plane oculomotor responses, including their addiative effect	13 healthy volunteers (6 female, mean age 25 years)	C-ETD (Chronos Inc., Berlin)	Binocular tracking<br><br>Horizontal and vertical eye movements<br><br>Torsional eye movements.	Stimuli: a central fixation point (0.32 cm in diameter) surrounded by 38 inclined white lines tilted at 45 degrees.<br><br>Statistical analysis:MANOVA, Shapiro-Wilk´s tests, SPSS	Wibble et al.
2020	Explore the feasibility of applying the SMI RED eye tracker bar to record eye movements in 10- and 11-year-old children while reading a text	33 subjects (19 aged 10 years, 14 aged 11 years) normally sighted children	SMI eye tracker (RED 250 m, SensoMotoric)	Reading time, reading speed, total number of saccades or fixations.	E-T benefits: Allows to recognize reading difficulties and monitoring treatment effects.<br><br>Limitations: the use of glasses and the sample size	Wertli et al.
2020	Evaluate the saccadic movements (SM) in Primary open angle glaucoma (POAG) patients and healthy controls during an exploratory VS digit-based task	7 POAG subjects<br><br>6 controls	Iscan ETL 100Hz (MA, USA)	Saccadic movements	Statistical analysis: Correlation test	Senger et al.
2020	Observe the eye movement behaviors in patients with cataract during performance-based tasks including visual search, face recognition and reading tasks.	30 Subjects with bilateral age-related cataract.<br><br>22 age-matched controls.	Tobii Pro X3-120 (Eye Tracker; Tobii AB Inc., Danderyd, Sweden)	Eye movements<br><br>Fixations	Prospective study.<br><br>Three tasks: visual search, face recognition, reading task.<br><br>Statistical analysis: paired Student's t-tests, Mann-Whitney U test, Spearman correlation. SPSS.	Wan et al.
2020	Test the effects of alternate occlusion flicker on symmetrical vergence responses to disparity steps.	-	Dual Purkinje image eye tracker	Vergence eye movements		Ramakrishnan & Stevenson.

2020	Assess whether the pattern and/or magnitude of fixational eye movements differed when a visual discrimination task was performed at the point of fixation versus in peripheral vision	14 subjects (9 females, mean age 38 +/- 7 years)	EyeLink 1000 Plus(SR Research, Ottawa, ON, Canada) at 1000 Hz	Fixations	Stimuli: Monocular tumbling E orientation discrimination task at three different eccentricities: 0° (fovea), 5°, and 10°	Raveendran et al
2020	Optical health analysis of visual comfort for bright screen display based on back propagation neural network	30 subjects (15 female)	Tobii X2–60 eye tracker	Pupil size<br><br>Blinks	Stimuli: target and three distractors simultaneously presented<br><br>Statistical analysis: paired t test, Pearson correlation coefficient	Wang et al.
2020	Assess amblyopic children's covert endogenous attention with a classical spatial-cueing paradigm and compared performance across eyes and between amblyopic and visually typical children	11 visually typical children<br><br>13 amblyopic children	Tobii4C eye tracker (Tobii Technology, Frankfurt am Main,Germany)	Paradigm: shape speed<br><br>Monocular central fixation	Stimuli: target and three distractors simultaneously presented<br><br>Statistical analysis: paired t test, Pearson correlation coefficient	Ramesh et al.
2020	Examine the recognition of novel and lexicalized compound words during sentence reading	26 subjects with normal or corrected to normal vision	Eyelink II (SR Research, Mississauga, Ontario, Canada) 500 Hz	Fixation duration<br><br>Gaze duration<br><br>First fixation duration<br>Selective regression-path duration	Stimuli: Sixty existing two-constituent compound words and 60 novel two-constituent Finnish compound words	Hyona et al.
2020	Examine the feasibility of saccadic vector optokinetic perimetry (SVOP), an automated eye tracking perimeter, as a tool for visual field (VF) assessment in infants	13 healthy infants (ae range 3.5 and 12 months)	Eye tracker inside Saccadic Vector Optokinetic Perimetry (SVOP)<br>Tobii IS-1 (TobiiTechnology, Stockholm, Sweden) 40-H	Eye position	Stimuli: circular spots with angular diameter of 0.43º, 0.86º and 1.72º<br><br>Test patterns of up to 40 points were used.	Perperidis et al.
2020	To describe, validate, and provide preliminary normative data for an open-source eye-movement perimeter (Eyecatcher)	64 normally sighted adults (43 females, mean 24.3 years)	Eyecatcher hardware with a Tobii EyeX (Tobii Technology, Stockholm, Sweden)	Fixations	Stimuli: 24-2 Threshold test, usingGoldmann III/0.43° stimuli<br><br>Statistical analysis : .MatlabMann-WhitneyUtest	Jones.

2020	The effect of refractive error on optokinetic nystagmus	Experiment 1: 20 participants (20-35 years old)<br><br>Experiment2.. 25 subjects (19-51 years old)	Eyelink 1000 Plus, SR Research, Ontario, Canada) 500 Hz	Eye position<br><br>Eye velocity<br><br>Saccades	Stimuli: t two-dimensional random noise patterns. 2 experiments.<br><br>Statistical analysis: Matlab	Doustkouhi et al.
2019	Study the Fixation instability during binocular viewing in anisometropic and strabismic children	160 children age 4–12 years with treated esotropia and/or anisometropia (98 amblyopic, 62 nonamblyopic) were compared to 46 age-similar control	Eyelink II (SR Research, Mississauga, Ontario, Canada) 500 Hz	Fixation stability	Distance: 115 cm.<br><br>Monocular calibration.<br><br>Statistical analysis: Matlab. Bivariate contour ellipse area to measure fixation stability. ANOVA	Kelly et al.
2019	Present a near-eye augmented reality display with resolution and focal depth dynamically driven by gaze tracking	60 people	Own wearable Display Prototype	-	Data analysis: paired t-test, independent t-test, simple correlation. SPSS was used.	Kim et al.
2019	Evaluate a smartphone application (app) performing an automated photographic Hirschberg test for measurement of eye deviations.	25 nonstrabismic normally-sighted subjects (age range 20- 40 years)	EyeTurn mobile app	Eye deviations in horizontal direction<br><br>Gaze angles	3 experiments	Pundlik et al.
2019	Effects of blue light on dynamic vision	16 ypung male adults (age range 25.9 years) with normal or corrected to normal binocular vision	Eyelink 2000. 1000 Hz SR Research,Mississauga, Ontario, Canad	Eye pursuit<br><br>Kinetic visual acuity<br><br>Dynamic visual acuity<br><br>fixation	Stimuli: A square (22.72° × 22.72°) in blue or orange as background was displayed during the whole experiment. Three randomly chosen numbers (0–9, size 1.89°) moved sequentially.<br><br>Statistical analysis: SPSS, ANOVA	Chen & Yeh.
2019	To detect eye tracking abnormalities in children with strabismus in the absence or presence of amblyopia	50 children with strabismus (24 with amblyopia) (mean age 10.66 +/-2.90 years)<br><br>50 controls<br><br>(mean age 10.02 +/- 2.75 years)	-	Paradigms:<br>1. Distance/near<br>2. Reading<br>3. Location Identification<br>4. Video	E-T benefits: detect eye movements deficits	Al- Haddad, C. et al.

				Metrics: number and time of fixations		
2019	Study interocular correlations of drifts when subjects fixated on a stationary target.	8 naïve subjects	A built high-resolution binocular eye tracker using two USB3 infrared monochrome cameras having 640x480 pixel resolution, and sampling images at 400 Hz (TheImagingSource, Model DMK33UX174)	Microsaccades	Stimuli: small yellow target on a black screen.<br><br>Distance 50 cm with chin rest.	Ivanchenko et al.
2019	Combining visual sensory functions and visuospatial orienting functions in children with visual pathology	119 children with visual impairment (age 1 to 12 years)	Tobii T60XL (Tobii Corporation, Danderyd, Sweden)	Reaction time to fixation<br><br>Fixation duration<br><br>Gaze fixation	Longitudinal study.<br><br>Paradigm: Preferential looking.<br><br>Stimuli: various visual stimuli, each was placed in one of four monitor quadrants.<br><br>Statistical analysis: Matlab	Kooiker et al.
2019	Study Presaccadic motion integration drives a predictive postsaccadic following response	8 participants (18-22 years old) with normal vision	Infrared eye tracker (USB-220, Arrington Research, Scottsdale, AZ)	Eye position	Distance: 95.5 cm<br><br>Stimuli: four equally eccentric motion dots.	Kwon et al.
2019	Test directly whether pursuit catch-up saccades and fixational micro-saccades exhibit the same temporal pattern of task-related bursts and subsidence	4 subjects	Eyelink 1000<br><br>1000 Hz	Fixations<br><br>Pursuit saccades<br><br>Saccades amplitude	Stimuli: created in Matlab.<br><br>Discrimination task, 15-character alphanumeric array paradigm, fixation, and pursuit task	Badler et al.
2019	Propose and evaluate a new approach to paracentral VF assessment that combines an inexpensive eye-tracker with a portable tablet computer (“Eyecatcher”)	24 eyes from 12 glaucoma patients<br><br>12 eyes from 6 controls	Eyecatcher<br><br>Tobii EyeX eye-tracker (Tobii Technology, Stockholm, Sweden) 50 Hz	fixations	Distance: 50 cm<br><br>Stimuli: programmed in C# and R. White Goldmann III (0.43°) circular spots, presented at a fixed intensity of 300 cd/m2	Jones et al.

			Without chinrest			
2019	Propose a compensatory visual-field training using game-like dynamic scenes presented by a head-mounted display eye tracker (HMD-ET)	10 subjects with normal or corrected to normal vision	HMD-ET. 240 Hz	Eye fixations during a compensatory visual fiel training	E-T permits to reveal differences in patterns of visual exploration between people	Hotta et al.
2019	Determine the latencies of orienting responses during a preferential looking task in children with normal vision and in children with visual impairments	Eighty-eight children (9.61.8 years) with normal vision (NV), 15 children(9.01.6 years) with cerebral visual impairment (CVI), and 19 children(9.02.4 years) with visual impairment due to congenital or acquired disorders of the eye without additional impairments	Stereoscopic eye tracking system with two USB 3.0 cameras and two infrared lights. 300 Hz	Gaze position<br><br>Saccades<br><br>Fixations<br><br>Fixation time	Paradigm: preferential looking.<br><br>Stimuli: 2 × 2 grid, with three uniform grey fields and one target field consisting of a black-and-white square wave grating.<br><br>E-T: provides objective detection measures	Barsingerhorn et al.
2019	Explore the problems associated with calibration and propose a method that secures a repeatable and reliable gaze estimation.	8 patients with nystagmus	EyeLink 1000 Plus. 1000 Hz	Calibration on targets		Rosengren et al.
2019	Confirm that visual crowding resulted in decreased performance and prolonged saccadic reaction time (SRT) in crowding trials compared to reference trials	25 subjects (age range 19-26 years)	Tobii T60XL<br><br>eye tracker (Tobii Corporation, Sweden)	Binocular eye movements	Paradigm: visual crowding<br><br>Distance: 57 cm.<br><br>Stimuli: 5 charts with different fixation stimulus, color, letter, contrast, spatial frequency and shape.<br><br>Statistical analysis: Matlab<br><br>Pilot study was applied first.	Pel et al.
2019	Describe the functional and activity-based outcomes from an intense vision therapy program targeting visual function issues, including issues related to peripheral visual field loss, following stroke	3 females subjects (29, 29 62 years old)	SMI eye tracker<br><br>250 Hz	Fixation, saccades, visual search and reading		Axelsson et al.

2019	Assess the visual function, reading performance, and compensatory head posture (CHP) in schoolchildren with infantile nystagmus	18 participants aged between 13 to 18 years old were divided into spectacle (n=9) and null zone group (n=9	Tobii TX300 eye tracker	Time of reading	Sample size: Cochran`s formula. Participants were recruited randomly.	Fadzil et al.
2019	Development of a virtual reality (VR) video game platform, guided through Fuzzy Logic, targeting casualties with amblyopia and convergence insufficiency conditions	Age range 9-18 years	FOVE (head-mounted display) 100 Hz.	Eye positions	Stimuli: games of Vision Labs	Esfahlani et al
2019	Study how restricting visual acuity of normally sighted subjects would affect visual search and navigation in a real-world environment, and how their performance would compare to subjects with naturally occurring low vision.	Experiment 1. 8 normally sighted subjects (mean age 23)<br><br>Experiment 2. 8 low vision subjects (mean age 55 years)	Tobii Glasses 1<br><br>mobile head-mounted eye tracker (Tobii Technology, Inc., Falls Church, VA., https://www.tobii.com/)	Fixation	Two experiments: looking for objects and comparison between acuity-restricted normally sigthed subjects and low vision people.<br><br>Statistical analysis: ANOVA	Freedman et al.
2019	Study task-driven visual exploration at the foveal scale	31 emmetropic human (age range 18-25 years)	Generation 6 Dual Purkinje Image (DPI) eye tracker (Fourward Technologies)	Microsaccades<br><br>Drifts<br><br>Saccades	Stimuli: images of faces taken from online databases	Shelchkova et al.
2019	Investigate whether and how visual acuity at selected foveal locations changes before the onset of microsaccades	6 emmetropic subjects (5 females, age range 19-29 years)	Generation 6 Dual Purkinje Image (DPI) eye tracker (Fourward Technologies)	Saccades<br><br>Microsaccades		Shelchkova & Poletti
2019	Study smooth pursuit of a modally completed images	10 participants with AMD (mean age 77.9 +/- 7.8)<br><br>12 control participants (mean age 44.5 +/- 20.5)	Eyelink 1000 (SR Research)	Saccadic eye movements	Stimuli: smooth pursuit	González et al.
2019	Investigate human interceptive saccades and pursuit responses to moving targets defined by high and low luminance contrast or by chromatic contrast only (isoluminance)	11 subjects (8 females, mean age 24.7 +/- 3.2 years)	EyeLink 1000 Plus (SR Research Ltd., Mississauga, Canada) 1000 Hz	Eye position<br><br>Saccades		Goettker et al.

2019	Study how rapidly infants detect human faces in complex naturalistic visual scenes	241 infants (3 months -12 months)	Eyelink 1000+ (SR Research, Ontario) 500 Hz	Fixations<br><br>Saccades	Stimuli: Visual Saliency. P<br><br>Photographs of complex indoor and outdoor scenes presented with Experiment Builder and data was extracted with Data Viewer.<br><br>Distance: 60 cm<br><br>Statistical analysis: Matlab	Kelly et al.
2019	Study eye Movements of Drivers with Glaucoma on a Visual Recognition Slide Test	31 older drivers with glaucoma (mean age 71.7 +/- 6.3 years)<br><br>25 age matched with normal vision (mean age 71.1 +/- 6.6. years)	Tobii TX300 (Tobii Technology, Danderyd ,Sweden)	Total fixations<br><br>Number of fixations per second<br><br>Average fixation duration<br><br>Average saccades amplitude<br><br>Horizontal and vertical variance	Stimuli: DriveSafe test.<br><br>Statistical analysys: R was used. Mann-Whitney U test, chi square test	Lee et al.
2018	Examine the characteristics of small saccades by means of two of the most established high-resolution eye-tracking techniques available	7 subjects<br><br>Observers<br><br>4 subjects (age range 30-70)	Binocular Dual Purkinje Image eye tracker<br><br>Revolving Field Monitor, a specially designed eye-coil apparatus<br><br>Eyelink 1000+ (SR Research, Ontario) 500 Hz	Fixations<br><br>Small saccades<br><br>Microsaccades<br><br>Saccades amplitude	Stimuli:small crosses (27′ × 2′ bars) displayed on a CRT monitor at a refresh rate of 200 Hz<br><br>Head-rest<br><br>Distance: 50-60 cm	Fang et al
2018	Characterize how acuity restriction affects gaze behavior while subjects with normal and low vision searched for and identified targets during navigation	8 low vision subjects<br><br>8 normally sighted controls	Tobii head-mounted eye tracking system	Gaze transitions<br><br>Gaze direction	Stimuli: look for objects<br><br>Statistical analysis: correlation tests	Freedman et al.

2018	To investigate whether glaucoma produces measurable changes in eye movements	50 patients with glaucoma and asymmetric vision loss	Eyelink 1000 (SR Research Ltd., ON, Canada)<br><br>1000 Hz	Saccadic reversal rate (SRR), saccade amplitude, fixation counts, fixation duration, and spread of fixation locations	Distance: 60 cm<br><br>Chin rest<br><br>Stimuli: viewed monocularly displayed duration between 3 and 5 seconds. 39 color images and 81 grayscale images, natural scenes.<br><br>Statistical analysis: Wilcoxon`s test. Multiple regression analysis.R was used.	Asfaw, D. , et al.
2018	Investigate the effects of a specially designed combat sports (CS) training program on the visuomotor performance levels of children	Children aged 9-12 years	EyeLink 1000 (SR Research Ltd., ON, Canada)<br><br>With chinrest	Saccades<br><br>Eye response time	Stimuli: hit or visually track.<br><br>Statistical analysis: comparative statistic, descriptive statistic	Ju et al.
2018	Compare the performance of an automated and objective method to measure near heterophoria using an eye-tracker with two conventional methods: the cover-uncover test and the modified Thorington test	30 non-presbyopic adults (15 females, mean ae 27.9 +/-4.6 years)	EyeLink100 250 Hz	Eye position<br><br>Fixation	Stimuli: fixation stimulus consisted of an empty black circle of 1.6˚.The inner white region subtended an angle of 0.9˚with a 20/50(0.21˚)Snellen E letter at the center to favor fine fixation.<br><br>E-T benefits: provides objective and more repeatable measures, should be the new gold standard to measure heterophoria.	Mestre et al.
2018	Determine the optimal gain (stimulus/eye motion ratio) that corresponds to maximum performance in an orientation-discrimination task performed at the fovea	7 human subjects with normal or corrected to normal vision (ages 18-35)	Tracking Scanning Laser Ophthalmoscope (TSLO; Shehhy et al., 2012)<br><br>Eye movement sampling rate of 960 Hz	Eye movements		Agaoglu, M. et al.
2018	Investigate the impact of training with action video games on balance function in older adults using a randomized controlled trial design.	146 healthy adults (age over 60)		Saccadic eye movements<br><br>Pursuit eye movements	Stimuli: 20 hours of cartoon-like action video-game training.<br><br>Pretest, training, postest	Cheong et al.

				Fixations		
2018	Test if a scanning mode utilizing eye movements increases visual stability and reduces head movements in Argus II users	8 blind individuals	Eye Tracking Glasses 2.0 (ETG 2.0; SensoMotoric Instruments, Teltow, Germany) 60 Hz	Location of the target	Stimuli: target that had to be touched.<br><br>E-T benefits: Integrating an eye tracker into the Argus II is feasible, reduces head movements in a seated localization task, and improves pointing precision.	Caspi et al.
2018	Examine whether the stability of steady fixation was different in individuals with corrected myopia and corrected hyperopia during a fixation task.	10 healthy subjects (mean age 24.2 +/- 2.6 years)	Eyelink 1000. 1000 Hz	Fixation stability	Monocularly<br><br>Vertical and horizontal eye position<br><br>BCEA<br><br>Statistical analysis: descriptive statistic	Coletta et al.
2018	Evaluate differences in eye movements during reading in strabismic children and in non-strabismic age-matched children, and to evaluate the potential effect of strabismus surgery on eye movement performance.	9 Strabismic children (age range 11- 15 years)	Mobile T2 (SuriCog, France) 300 Hz	Fixations<br><br>Fixations duration	Stimuli: reading task with both eyes and monocularly.	Perrin-Fievez et al.
2018	Develop a tracking test to record participants' eye-movements while we simulated different gaze-contingent Visual Field Defects.	50 healthy participants and 1 glaucoma patient	Eyelink 1000 (SR-Research, Kanata,Ontario, Canada)	Saccadic pursuit<br><br>Smooth pursuit	Stimuli: Gaussian luminance blob (FWHM = 0.5 degrees of visual angle) moving in a2D random-walk path.	Grillini et al.
2018	Automatic Detection of Preferred Retinal Locus (PRL) for Low Vision Rehabilitation using Oculometrics Analysis	9 participants with normal vision	Tobii Pro TX-300 (Tobii AB) 300 Hz	Fixations	Statistical analysis: Median Absolute Deviation.<br><br>E-T benefits: it allows an automated PRL detection system.	Yow et al.
2018	Develop a comprehensive protocol for suction stability management during small incision lenticule extraction (SMILE)	-	-	Fixation light tracking, Bell's reflex, saccades, oscillations, nociceptive reflex movement, false	Stimuli: video recording during incision.	Reinstein et al.

				suction, and nystagmus		
2018	Characterize the gaze stability of young infants, adult participants and 4- to 10-week-old infants	22 typically developing, full-term infants (4-10 weeks) and 13 adults	EyeLink 1000 (SR Research, Ottawa, Canada) 250 Hz	Fixations<br><br>Stability of gaze position<br><br>First visit	Stimuli: Random-noise patterns were presented on a rear-projection screen that preserved circular polarization. Monocularly. Psychophysics Toolbox<br><br>Statistical analysis: Shapiro-Wilks test, Welch`s test	Seemiller & Candy.
2018	Use standard automated perimetry to compare fixation variability among the dominant eye fixation, non-dominant eye fixation, and binocular fixation conditions	35 healthy eyes of 35 subjects	Tobii glass II (TobiiTechnology, Stockholm, Sweden) 50 Hz	Fixation variability	Cross-sectional study<br><br>BCEA<br><br>Statistical analysis: R was used, paired t-test, Pearson`s correlation, Bonferroni test	Hirasawa et al.
2018	Explore the relationship between eye movements performance and Visual Acuity, central Visual Field, as well as AMD features	187 subjects	Tobii TX300	Gaze points	Cross-sectional study.<br><br>Monocularly.<br><br>Statistical análisis: Student`s t test. Linear regression analysis. Significance level 0.05<br><br>E-T benefits: Tracking eye gaze can assist visual function assessment in AMD.	Laude et al.
2018	Describe an efficient new tool for measuring contrast sensitivity, Curveball, and empirically validate it with a sample of healthy adults	35 healthy adults (19 women, 16 men; mean age 38.6615.8) with normal or corrected-to-normal	Tobii 4C eye tracker (Tobii Technology, Stockholm, Sweden	Smooth tracking	Stimuli: curve ball task	Mooney et al.
2018	Assess the extent of visual search deficits in amblyopia using feature and conjunction search tasks.	10 participants with anisometropic, strabismic, or mixed amblyopia	EyeLink 1000 remote eye tracker (SR Research, Ottawa, Canada	Fixations<br><br>Saccades	Distance: 100 cm<br><br>Chinrest<br><br>Stimuli run with Psychotoolbox with Matlab. Four experiments: contrast threshold estimation, target separation estimation, feature search, conjunction search.	Tsirlin et al.

					Statistical analysis: ANOVA, Welch`s test, alpha 0.05.	
2018	Propose a new (micro)saccade detection method, based on an unsupervised clustering approach, that can effectively separate fixational microsaccades from high-frequency recording noise	14 subjects (age range 69-81 years)	The EyeLink 1000 (SR Research Ltd., Ontario, Canada)	Microsaccades	Distance: 57 cm<br><br>Monocularly	Sheynikhovich et al.
2018	Investigate how peoples' eye movements adapt and optimize in response to a simulated scotoma in an object recognition task using a gaze-contingent display.	10 participants (4 males, mean age=26.2y) with normal visual acuity	Tobii PRO TX-300	Eye movements<br><br>Saccades<br><br>Fixations	Stimuli, indicate wether a randomly placed object was either a non-sense object or a known one.120 trials per session.<br><br>Distance: 60 cm<br><br>Chinrest<br><br>Training with 9 sessions, two per weel.	Ryu & Wallraven
2017	Develop a novel dichoptic optokinetic nystagmus (OKN) paradigm and investigate its effectiveness in objectively quantifying the interocular suppression in subjects with monocular amblyopia	8 amblyopia subjects (amblyopia group) and 8 age- and sex-matched normal subjects (control group)	EyeLink (SR-Research, Ontario,Canada)	Eye movements<br><br>Blinks	Distance: 57 cm<br><br>Statistical analysis: Correlation. Spearman`s correlation.	Wen et al.
2018	Measure cone-sampling-limited acuity at sub-foveal intervals and measure the functional role of microsaccades (MS) when presented with at-threshold stimuli at varying sub-foveal distances from the preferred fixation locus (PRL).	3 males and 3 females (ages 25-29 years) with no known visual issues	Adaptive optics scanning laser ophthalmoscope (AOSLO)	Fixational eye movements	Stimuli: tumbling-E	Ratnam et al.
2018	Determine the ability of Saccadic Vector Optokinetic Perimetry (SVOP) to detect and characterize visual field defects in children with brain tumors using eye-tracking technology	16 patients (mean age 7.2 years, 7 male)	IS-1 eye tracker<br><br>X50 eye tracker	Eye gaze<br><br>Fixations	Stimuli. Fixation tasks	Murray et al.

				Direction and amplitude of each saccade		
2018	Evaluate binocular eye movements to targets when central vision is compromised. Study if binocular vision deficits may be underestimated by monocular vision tests and identify a method that can be used to select a PRL based on binocular contrast summation	10 participants<br><br>(Mean age 24.1 years)	Eyelink II (SR Research, Ottawa, Canada)<br><br>500 Hz	Location of each eye	Stimuli: generated and presented with Matlab. Full alphabet<br><br>Distance: 50 cm<br><br>Statistical analysis: parametric analysis, R was used. Bonferroni test for multiple comparisons.	Alberti & Bex.
2018	Study the miniature eye movement for seeing fine spatial details	7 subjects (range age 18-35 years) with normal or corrected vision	Tracking Scanning Laser Ophthalmoscope (TSLO; Shehy et al., 2012)	Fixaional eye movements	Stimuli: task was to report the orientation of a sinusoidal grating from vertical. Dark room,	Ağaoğlu et al.
2017	Investigate whether the normal aging process influences the ability to adapt disparity vergence and phoria	49 healthy subjects<br><br>(Ages 20-70 years)	Video-based ISCAN eye movement monitor<br><br>LabVIEW™ program, VisualEyes, with 12-bit digital acquisition hardware card<br><br>500Hz	Eye movements, tracking both eyes simultaneously and independently<br><br>Peak velocity	Distance: 40 cm<br><br>Chin rest<br><br>Statistical analysis: Matlab, ANOVA, linear regression analysis.Pearson correlation.	Alvarez, T. et<br><br>al.
2017	Assess if adaptations improve binocular contrast sensitivity in the peripheral visual field	6 subjects normally sighted obervers	Eyelink II 100 Hz	Fixation	Stimuli: 26AFC task. Letters were positions 2º in the lower visual field.	Alberti & Bex
2017	Characterize saccadic rhythmicity, and examine whether it is consistent with an autonomous oscillatory generator or with a self-paced generation	36 students with normal vision<br><br>First experiment: 12 subjects, 7 females (mean age 25.9 +/-2.3 years)	Eyelink 1000 Plus<br><br>SR Research, Canada<br><br>1000 Hz	Saccades<br><br>Microsaccades	Stimuli: An 11 minutes long nature movie clip with sound. 3 experiments.<br><br>Distance: 57 cm	Amit, R., et al.

		Second experiment:11 subjects, 6 females (mean age 26-1 +/- 2.7 years)<br><br>Third experiment: 12 subjects, 7 females (mean age 26.1 +/- 2.7 years)				
2017	Perform usability testing of a binocular optical coherence tomography(OCT) prototype to predict its function in a clinical setting.	45 participants with chronic eye disease (mean age 62.7 years)<br><br>15 healthy controls (mean age 5 years)	Optical coherence tomography (OCT)	Fixations	Monocularly.<br><br>Stimuli: fixation target in primary position and 8 positions of gaze 4º from center.	Chopra et al.
2017	Determine if microsaccades are altered in hemianopia; how altered microsaccade features correlate with visual performances; and how their direction relates to visual field defect topography.	14 hemianopic stroke patients (13 male, mean age 59)<br><br>14 healthy controls (11 male, mean age 60)	EyeLink-1000 system (SRResearch, Ontario, Canada) 500 Hz	Microsaccades (rate, amplitude, and velocity)	Case-control study<br><br>Stimuli: fixation dot in 41 trials lasting 7 secons each one	Gao & Sabel.
2017	Explore the effect of latency for foveated rendering in Virtual Reality (VR) applications and evaluate the detectability of visual artifacts for three techniques capable of generating foveated images and for three different radii of the high-quality foveal region	First experiment: 9 subjects<br><br>Second experiment:<br><br>2 subjects	SMI eye tracker<br><br>250 Hz<br><br>Tobii TX300 eye and head tracker<br><br>300 Hz	Metrics: head translation, head rotation, frequency of saccades, saccades amplitudes	Stimuli: classroom scene	Albert, R., et al.
2017	Investigates whether the induced PRLis transferred to important visual tasks in daily life, namely pursuit eye movements, signage reading, and text reading	50 subjects. 10 females (mean age 26.6 years)	Eyelink 1000 Plus. 1000 Hz	Saccades velocity<br><br>Pursuit eye movements	Simulate scotoma<br><br>Chinrest<br><br>Distance: 62 cm<br><br>3 sessions: saccades task, pursuit task, signage reading, text reading.	Barraza et al

2017	Evaluate whether the eye tracking system (ETS) improved the reproducibility of a single circle peripapillary retinal nerve fiber layer (RNFL) measurement acquired with spectral-domain optical coherence tomography (OCT)	205 people<br><br>100 healthy individuals<br><br>105 patients with open-angle glaucoma	Spectralis self-acting eye-tracking (eye tracker)	Eye movements	Statistical analysis: correlation coefficient, coefficient of variation, test-retest variability.	Abadia, B,. et al.
2017	Investigate the impact of accommodation, convergence, and proximity on the pupillary diameter.	12 subjects (6 female, age range 21-28)<br><br>Experiment 2: 9 subjects (2 female, mean age 25 years)	EyeLink 1000 with Experiment Builder (V1.10.165, SR Research Ltd., Mississauga, Ontario, Canada)	Pupil size<br><br>Pupil response<br><br>Vergence eye movements	Chin rest<br><br>Distance: 25 cm and 4 m<br><br>Stimuli: Landolt rings as targets<br><br>Statistical analysis: ANOVA, SPSS, one sample t test	Feil et al.
2017	Characterize eye movements made by patients with intermittent exotropia when fusion loss occurs spontaneously and compare them with those induced by covering 1 eye and with strategies used to recover fusion	13 patients with typical findings of intermittent exotropia who experienced a frequent spontaneous loss of fusion (6 male, range 11-61 years)	Infrared video-based eye tracker (iView X, SensoMotoric Instruments), sampling at 60 Hz	Eye position<br><br>Peak velocity	Calibrated monocularly.<br><br>Chinrest<br><br>Accepted ± 1º accuracy<br><br>Distance: 57 cm<br><br>Stimuli: central target and peripheral target in random location.<br><br>Data analysis: Wilcoxon rank sum test	Economides & Adams
2017	Investigate the relation between the placement of sustained attention and the location of a developed PRL using simulations of central scotoma	30 subjects (4 males, mean age 25.3 years)	Eyelink 1000. 100 Hz	Fixation :stability, eccentric<br><br>Saccades	Stimuli: Matlab, Psychotoolbox and Eyelink toolbox were used. Target was located along different meridians, 8 locations, 1 second each target. Scotoma was simulated.<br><br>Chinrest<br><br>Distance: 66.6cm<br><br>Statistical analysis: BCEA,	Barraza et al.

2017	Determine if the deviation angle changes in subjects with intermittent exotropia they alternate fixation between the right and left eye in primary gaze	37 subjects with intermittent exotropia	iViewX (SensoMotoric Instruments, Teltow, Germany)	Eye positions	Calibrated monocularly<br><br>Chinrest<br><br>Accepted ± 1º accuracy<br><br>Distance: 57 cm<br><br>Stimuli: central target and peripheral target in random location, people had to saccade to the peripheral target<br><br>Data analysis: blinks were excised, mean position and SD. Wilcoxon rank sum test.	Adams et al.
2017	Assess whether such adaptations improve binocular contrast sensitivity in the peripheral visual field.	6 subjects with normally sighted	Eyelink II<br><br>1000 Hz	Fixation control	Stimuli: letters were positioned 2º in the lower visual field. Controlled with nVidia 3D glasses	Concetta, A. & Bex, P.
2017	Study if Binocular Fixation Reduces Fixational Eye Movements in the Worst Eye of Patients with Center-Involving Diabetic Macular Edema	57 eyes from 29 diabetic patients	iView X™ video-based eye tracker	Monocular and binocular fixations Fixation stability (BCEA)<br><br>Minimum saccade velocity<br><br>Fixational saccades	Cross-sectional study<br><br>Chinrest and foreheadrest<br><br>Stimuli: black cross was presented for 40s.<br><br>Statistical analysis: student paired t test, Bonferroni multiple comparison tests, simple linear regression to correlations	Jakobsen et al.
2017	Study the Impact of eye tracking technology on OCT-Angiography imaging in patients with age-related macular degeneration	30 eyes of 30 AMD patients (21 females, aged78.97 ± 9.7 years, range 58–94 years)	OCT-system (AngioVue, RTVue XR Avanti SD-OCT, Optovue,Fremont, CA, USA)	Fixations<br><br>Blimks	Statistical analysis: Kolmogorov-Smirnov. Non-parametric tests. Coefficients of variation. Spearman r test. Wilcoxon test (paired) and Mann-Whitney test (unpaired). Statistical significance at 0.05<br><br>E-T benefits: improves image quality in OCT-A	Lauermann et al.
2017	Assess the eye movement patterns and visual predictors of performance on a	30 older drivers with glaucoma (71±7 years) and 25 age-matched controls (72±7 years).	Tobii TX300eye-tracker (Tobii Technology, Danderyd, Sweden),	Response time<br><br>Smaller saccades	Stimuli: Hazard Perception Test	Lee et al.

	laboratory-based hazard detection task in older drivers with glaucoma.			First fixations	Statistical analysis: SPSS, level of significance 0.05. Independent sample t-test. Linear mixed-effects models.	
2017	Develop a method can be used to detect and characterize sensorimotor deficits associated with TBI	34 Traumatic Brain Injury subjects	Comprehensive Oculometric Be-havioral Response Assessment (COBRA)	Pursuit latency<br><br>Pursuit acceleration<br><br>Saccades amplitude<br><br>Smooth tracking	Stimuli: 15 minutes eye movement tracking, 180 trials. Comprehensive Oculometric Behavioral Assessment (COBRA)<br><br>Chinrest	Liston & Stone.
2017	Study if patients with AMD would be able to track both the completed and visible pursuit stimuli by using peripheral retinal information.	4 patients with AMD and 11 controls with normal vision	Tobii TX300	Smooth pursuit<br><br>Mean peak velocity gain<br><br>Saccade frequency<br><br>Sacadde amplitude		Liu et al.
2017	Detect AMD caused vision impairment from gaze data	74 eyes of 57 Patients with AMD	Tobii TX300<br><br>Nidek Microperimetry	Fixations<br><br>Smooth pursuit	Monocularly<br><br>Distance. 60 cm	Liu et al.
2017	Analyze the differences between deviations of both eyes and the displacement of one single eye during the measurement of horizontal phoria in the cover test	9 subjects (mean age 22.3 ± 3.5 years), normal or corrected-to-normal visual acuity and a horizontal phoria greater than 1 PD	Eye-tracker embedded in the stereoscopic virtual reality system EVA (Eye and Vision Analyzer, Davalor Salud, Spain)	Eye position	Distance: 40 cm<br><br>Stimuli: Fixated an stimulus during 2 cycles: binocular vision, right eye occlusion, binocular vision, left eye occlusion.<br><br>Statistical analysis: dependent t-test, means and SD<br><br>E-T benefits: the possibility of registering both eyes`s movements.	Mestre et al.
2017	Investigate the relationship between silent reading performance and visual	50 glaucoma patients (mean age,52.2 +/-11.4years)and 20 normal controls(mean age,46.9 +/-17.2 years)	Tobii TX300 (Tobii Technology Danderyd, Sweeden) 300 Hz	Reading duration	Prospective cross-sectional study	Murata et al.

	field defects in patients with glaucoma using an eye tracking			Mean fixation duration.<br><br>Total visit duration<br><br>Fixation count	Stimuli: 3 articles composed of 607-612 characters with 12-13 lines per paragraph<br><br>Distance: 60 cm<br><br>Environmental conditions were controlled.<br><br>Statistical analysis: SPSS, Mann-Whitney U test, chi-square test, student`s t test. Spearman`s rank correlation coefficient.	
2017	Evaluate threshold saccadic vector optokinetic perimetry (SVOP) and compared results to standard automated perimetry (SAP)	162 subjects (103 with glaucoma and 59 healthy subjects)	Tobii IS-1 model (Tobii Technology, Stockholm, Sweden)	Eye gaze responses<br><br>Fixation<br><br>Eye location	Cross-sectional study<br><br>Stimuli: a fixation target<br><br>Distance: 55 cm<br><br>Statistical analysis: Shapiro-Wilk tests, Wilcoxon rank-sum test, ANOVA. SPSS. Level of significance 0.05.	Murray et al.
2017	Examine the relation between dynamic visual acuity (DVA) and the kinematics of smooth pursuit and saccadic eye movements	23 males (mean age19.5 +/- 1.2 years)	Eyelink 1000 Desktop Mount (SR Research Ltd., Ottawa, ON, Canada) 1000Hz	Smooth pursuit<br><br>Eye position<br><br>Velocity<br><br>Saccades	Stimuli: computer-based dynamic object task, black Landolt-C rings.<br><br>Distance: 71.5 cm<br><br>Statistical analysis: ANOVA, bivariate correlation coefficients. Level of significance 0.05. SPSS	Palidis et al.
2017	Investigate reading rates in age-matched normal and early to intermediate AMD patients with similar acuity.	21 subjects (11 AMD, age 78.4 +/- 7.49)<br><br>(1o controls, ae 75.2 +/-5.75)	Tobii TX300 Eye Tracker	Saccades, fixations, regressions, and blinks	Stimuli: Wilkins reading test<br><br>Distance: 60 cm	Ridder et al.
2017	Investigate potential differences in comprehension, brain wave activity, and eye movements, when people read texts with or without blue-filtering lenses	34 participants (24 males, students, mean age=23.5y) with normal visual acuity	Tobii XL120, 120Hz	Eye movements<br><br>Saccades	Stimuli: 6 texts with different topics displayed for 120 s.<br><br>Statistical analysis: non-parametric tests. Matlab	Ryu & Wallraven

2017	Compare different metrics and acquisition modes of fixation stability as a new visual function biomarker in patients with ABCA 4-related Stargardt disease	235 patients	Nidek MP-1 microperimeter (Navis, Nidek Technologies, Italy)	Fixation stability(BCEA)<br><br>Fixations	Stimuli: a fixation target<br><br>Monocularly<br><br>Statistical analysis: correlation tests	Schönbach et al.
2017	Study monocular and Binocular Smooth Pursuit in Central Field Loss	7 participants with central field loss (ages: 52–91, 4 males) and 4 controls (ages: 70–84, 1 male)	Eyelink 1000	Smooth pursuit	Distance: 1m<br><br>Chinrest<br><br>Stimuli: 90 trials of the same viewing condition: binocular, monocular left and monocular right. White target that appeared at one of six locations<br><br>Statistical analysis: linear mixed-effects models. Correlation coefficients.	Shanidze et al.
2017	Explore whether character identification and image localization could be achieved through direct multiple-electrode stimulation with a suprachoroidal retinal prosthesis.	-	External infrared eye-tracking camera (Arrington Research,Inc., Scottsdale, AZ, USA	Eye movements<br><br>Gaze location	Stimuli: static imagen localization and dynamic imagen localization.<br><br>Statistical analysis: ANOVA.<br><br>E-T benefist: analyze the impact of eye movements on a performance in a task.	Shivdasani et al.
2017	Examine whether viewing distance affects fixation stability during binocular and monocular viewing	30 patients with AMD	video-based eye-tracker (Series 2020; El-Mar, Inc., Toronto, ON, Canada) 120 Hz	Fixations BCEA<br><br>Blinks<br><br>Saccades	One experimental session<br><br>Chinrest<br><br>Distances: 40 cm, 1 m and 6m<br><br>Monocularly and binocularly<br><br>Statistical analysis: Shapiro-Wilk test, ANOVA	Tarita et al.
2017	How crowding and collisions affect gaze anchoring and dual-task performance	14 subjects (7 female, age range 20.4 +/- 1.1 years)	Monocular eye-tracking system, EyeSeeCam, 220 Hz (ESC; EyeSeeTech GmbH, Furstenfeldbruck, Germany)	Paradigm:<br><br>Multiple-object tracking.	Stimuli: Matlab, 10 stationary squares. 20 test blocks grouped 12 test trials.<br><br>2 sessions	Vater et al.

				Vertical and horizontal rotations of the right eye	Statistical analysis: ANOVA. Level of significance p=0.05	
2017	Determine the extent to which eye movements in children with delayed reading skills are different from those obtained from children with good/average reading skills in non-reading-related tasks	120 children without delayed reading skills and 43 children with delayed reading skills	Tobii TX300 eye tracker.	Fixation stability. Saccadic main sequences, and the number and amplitude of the saccades during fixation	Distance: 65 cm<br><br>Stimuli: animal cartoons that appeared in 4 positions or in the center of the screen<br><br>Statistical analysis: Matlba, SPSS, Shapiro-Wilk tests, parametric and no parametric tests, ANOVA, chi-square test. Mann-Whitney test.	Vinuela et al.
2017	Automatic Visual Impairment Detection System for Age-related Eye Diseases through Gaze Analysis	69subjects with clinically diagnosed AMD	Tobii Pro TX-300 (Tobii AB) 300 Hz	Gaze tracking	Stimuli: impulse stimulus response test and pursuit stimulus response. Monocularly	Yow et al.
